# The Evolutionary Dynamics of the Lion *Panthera leo* Revealed by Host and Viral Population Genomics

**DOI:** 10.1371/journal.pgen.1000251

**Published:** 2008-11-07

**Authors:** Agostinho Antunes, Jennifer L. Troyer, Melody E. Roelke, Jill Pecon-Slattery, Craig Packer, Christiaan Winterbach, Hanlie Winterbach, Graham Hemson, Laurence Frank, Philip Stander, Ludwig Siefert, Margaret Driciru, Paul J. Funston, Kathy A. Alexander, Katherine C. Prager, Gus Mills, David Wildt, Mitch Bush, Stephen J. O'Brien, Warren E. Johnson

**Affiliations:** 1 Laboratory of Genomic Diversity, National Cancer Institute, Frederick, Maryland, United States of America; 2 CIMAR, Centro Interdisciplinar de Investigação Marinha e Ambiental, Universidade do Porto, Porto, Portugal; 3 Laboratory of Genomic Diversity, SAIC-Frederick, Inc., NCI-Frederick, Frederick, Maryland, United States of America; 4 Department of Ecology, Evolution, and Behavior, University of Minnesota, St. Paul, Minnesota, United States of America; 5 Tau Consultants, Maun, Botswana; 6 Wildlife Conservation Research Unit, Tubney, Oxon, United Kingdom; 7 Laikipia Predator Project, Museum of Vertebrate Zoology, University of California Berkeley, Berkeley, California, United States of America; 8 Ministry of Environment and Tourism, Windhoek, Namibia; 9 Department of Wildlife and Animal Resources Management, Makerere University, Kampala, Uganda; 10 Uganda Wildlife Authority, Kamwokya, Kampala, Uganda; 11 Department of Nature Conservation, Tshwane University of Technology, Pretoria, South Africa; 12 Wildlife Veterinary Unit, Department of Wildlife and National Parks, Kasane, Botswana; 13 Department of Pathology, Microbiology, and Immunology, School of Veterinary Medicine, University of California Davis, Davis, California, United States of America; 14 SANParks, Endangered Wildlife Trust and Mammal Research Institute, University of Pretoria, Skukuza, South Africa; 15 Smithsonian's National Zoological Park, Conservation & Research Center, Front Royal, Virginia, United States of America; INRA, France

## Abstract

The lion *Panthera leo* is one of the world's most charismatic carnivores and is one of Africa's key predators. Here, we used a large dataset from 357 lions comprehending 1.13 megabases of sequence data and genotypes from 22 microsatellite loci to characterize its recent evolutionary history. Patterns of molecular genetic variation in multiple maternal (mtDNA), paternal (Y-chromosome), and biparental nuclear (nDNA) genetic markers were compared with patterns of sequence and subtype variation of the lion feline immunodeficiency virus (FIV_Ple_), a lentivirus analogous to human immunodeficiency virus (HIV). In spite of the ability of lions to disperse long distances, patterns of lion genetic diversity suggest substantial population subdivision (mtDNA Φ_ST_ = 0.92; nDNA *F*_ST_ = 0.18), and reduced gene flow, which, along with large differences in sero-prevalence of six distinct FIV_Ple_ subtypes among lion populations, refute the hypothesis that African lions consist of a single panmictic population. Our results suggest that extant lion populations derive from several Pleistocene refugia in East and Southern Africa (∼324,000–169,000 years ago), which expanded during the Late Pleistocene (∼100,000 years ago) into Central and North Africa and into Asia. During the Pleistocene/Holocene transition (∼14,000–7,000 years), another expansion occurred from southern refugia northwards towards East Africa, causing population interbreeding. In particular, lion and FIV_Ple_ variation affirms that the large, well-studied lion population occupying the greater Serengeti Ecosystem is derived from three distinct populations that admixed recently.

## Author Summary

The lion *Panthera leo*, a formidable carnivore with a complex cooperative social system, has fascinated humanity since pre-historical times, inspiring hundreds of religious and cultural allusions. Here, we use a comprehensive sample of 357 individuals from most of the major lion populations in Africa and Asia. We assayed appropriately informative autosomal, Y-chromosome, and mitochondrial genetic markers, and assessed the prevalence and genetic variation of the lion-specific feline immunodeficiency virus (FIV_Ple_), a lentivirus analogous to human immunodeficiency virus (HIV) that causes AIDS-like immunodeficiency disease in domestic cats. We compare the large multigenic dataset from lions with patterns of genetic variation of the FIV_Ple_ to characterize the population-genomic legacy of lions. We refute the hypothesis that African lions consist of a single panmictic population, highlighting the importance of preserving populations in decline rather than prioritizing larger-scale conservation efforts. Interestingly, lion and FIV_Ple_ variation revealed evidence of unsuspected genetic diversity even in the well-studied lion population of the Serengeti Ecosystem, which consists of recently admixed animals derived from three distinct genetic groups.

## Introduction

**Figure 1:**
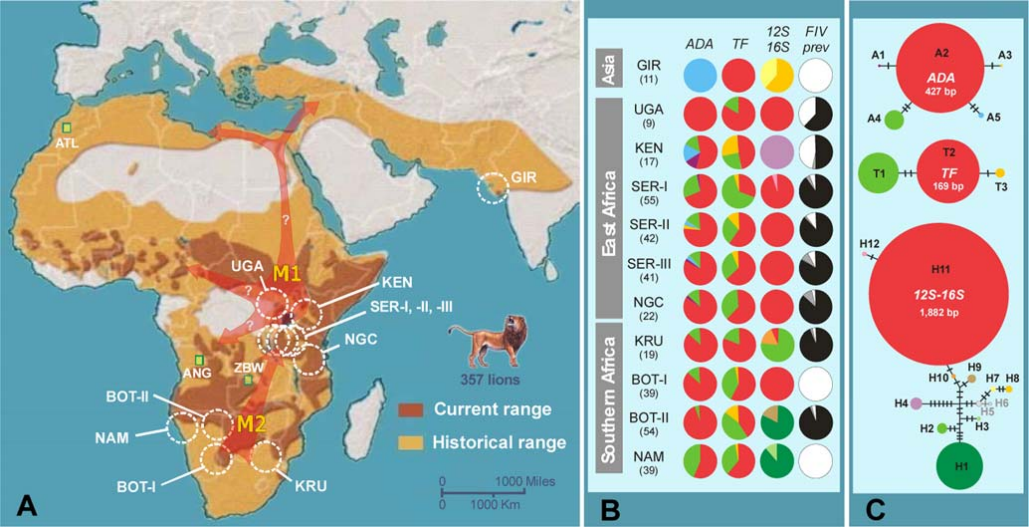
Geographic location of the lion samples and the variability of host and viral genetic markers among lion populations. (A) Historical and current geographic distribution of lion, *Panthera leo*. A three-letter code pointing to a white dotted circle represents the geographic location of the 11 lion populations determined by Bayesian analyses [22] and factorial correspondence analyses [23] of the genetic distinctiveness of 357 lion samples (see text): GIR, Gir Forest, India; UGA, Uganda (Queen Elizabeth National Park); KEN, Kenya (Laikipia), SER, Serengeti National Park, Tanzania; NGC, Ngorongoro Crater, Tanzania; KRU, Kruger National Park, South Africa; BOT-I, southern Botswana and Kalahari, South Africa; BOT-II, northern Botswana; and NAM, Namibia. Green squares represent captive individual samples to explore the relationship of lions from more isolated/endangered/depleted areas: ATL, Morocco Atlas lions (*n* = 4); ANG, Angola (*n* = 2); and ZBW, Zimbabwe (*n* = 1). Deduced historical expansions (M1 and M2) are represented by red arrows (see text). (B) Haplotype frequencies observed in the 11 lion populations for nDNA (*ADA* and *TF*), and mtDNA (*12S*–*16S*) genes, paralleled with the FIV_Ple_ serum-prevalence frequencies (black – sero-positive; gray – indeterminate; white – sero-negative). Population sample sizes are indicated within parenthesis. (C) Statistical parsimony networks of lion *ADA*, *TF*, and *12S*–*16S* haplotypes. Circle size is proportional to the haplotype frequency and crossbars represent the number of step mutations connecting haplotypes. The mtDNA haplotypes H5 and H6 are shaded gray as they were detected only in the individual samples from ANG, ATL, and ZBW, which do not group in unique population clusters (see text).

Lion fossils trace to the Late Pliocene in Eastern Africa and the Early Pleistocene in Eastern and Southern Africa coincident with the flourishing of grasslands ∼2–1.5 million years ago [1],[2]. By Mid Pleistocene (∼500,000 years ago), lions occupied Europe and by the Late Pleistocene (∼130,000–10,000 years ago) lions had the greatest intercontinental distribution for a large land mammal (excluding man), ranging from Africa into Eurasia and the Americas [3]. Lions were extirpated from Europe 2,000 years ago and within the last 150 years from the Middle East and North Africa. Today, there are less than 50,000 free-ranging lions [4] that occur only in sub-Saharan Africa and the Gir Forest, India (Figure 1A).

Understanding the broader aspects of lion evolutionary history has been hindered by a lack of comprehensive sampling and appropriately informative genetic markers [5]–[9], which in species of modern felids requires large, multigenic data sets due to its generally rapid and very recent speciation [10],[11]. Nevertheless, the unique social ecology of lions [12]–[14] and the fact that lions have experienced well-documented infectious disease outbreaks, including canine distemper virus, feline parvovirus, calicivirus, coronavirus, and lion feline immunodeficiency virus (FIV_Ple_) [15]–[18] provide a good opportunity to study lion evolutionary history using both host and virus genetic information. Indeed, population genetics of transmitted pathogens can accurately reflect the demographic history of their hosts [19],[20]. Unlike other of the 36 cat species, lions have a cooperative social system (prides of 2–18 adult females and 1–9 males) and their populations can have high frequencies of FIV_Ple_, a lentivirus analogous to human immunodeficiency virus (HIV), which causes AIDS-like immunodeficiency disease in domestic cats. FIV_Ple_ is a retrovirus that integrates into the host genome and is transmitted by cell-to-cell contact, which in felids occurs during mating, fighting and mother-to-offspring interactions. Thus, viral dissemination is a function in part of the frequency of contact between infected and naïve lions within and among populations. The virus is quite genetically diverse in lions [15],[18], offering a unique marker for assessing ongoing lion demographic processes.

To unravel lion population demographic history we used a large multigenic dataset. Distinct sets of markers may not necessarily yield similar inferences of population history, as coalescent times vary as a function of their pattern of inheritance [21]. There is also a large variance in coalescent times across loci sharing a common pattern of inheritance especially in complex demographical histories (Table 1). However, the accurate interpretation of the differences among loci can provide a more resolved and coherent population history, affording more-nuanced insights on past demographic processes, levels of admixture, taxonomic issues, and on the most appropriate steps for effective conservation and management of remaining populations.

**Table 1:** Expected and observed coalescent times for the different markers studied in lions according to their pattern of inheritance.

DNA region	Pattern of inheritance	Expected coalescence time in generations	Size (bp)	Number of haplotypes	*F*_ST_	Divergence Ppa/Ple (H)	Substitution rate (site/yr)	TMRCA (Myr ago)
mtDNA	Maternal haploid	2*N*_eff_≈*N*_ef_						
*12S and 16S*			1.882	12	0.92	0.0161	8.1×10^−8^	0.324
Y-Chromosomal DNA	Paternal haploid	2*N*_efm_≈*N*_ef_						
*SRY-3′*			1.322	1	_	0.0005	0.25×10^−8^	_
Autosomal DNA	Bisexual diploid	4*N*_ef_						
*ADA*			427	5	0.20	0.0023	1.2×10^−8^	4
*TF*			169*	3	0.09	0.0030*	1.5×10^−8^	4.8
*microsatellites*			na	na	0.18	na	2.1×10^−3^	0.027

TMRCA - Time to the most recent common ancestral haplotype.

*N*_ef_ - long-term inbreeding effective size of the population; *N*_eff_ - inbreeding effective size of females; and *N*_efm_ - inbreeding effective size of males.

The approximations to the expected coalescence time are made under the assumption that *N*_eff_ = *N*_efm_ = *N*_ef_/2.

*: The *TF* gene segment used to estimate the divergence Ppa/Ple was 450 bp long.

na - not aplicable.

The goal of this study was to assess the evolutionary history of lion by (1) characterizing lion population structure relative to patterns of FIV_Ple_ genetic variation, (2) detect signatures of migration using both host and viral population genomics, and (3) reconstruct lion demographic history and discuss its implication for lion conservation. We assess genetic variation from 357 lions from most of its current distribution, including mitochondrial (mtDNA; *12S*–*16S*, 1,882 bp), nuclear (nDNA) Y-chromosome (*SRY*, 1,322 bp) and autosomal (*ADA*, 427 bp; *TF*, 169 bp) sequences, and 22 microsatellites markers. We further document patterns of FIV_Ple_ variation in lions (FIV_Ple_ *pol*-RT gene, up to 520 bp).

## Results/Discussion

### Population Structure of Lion

Genetic analyses of 357 lions from throughout the extant species range showed that paternally inherited nDNA (*SRY*) and maternal inherited (mtDNA) sequence variation was generally low (only one paternal *SRY*-haplotype and 12 mtDNA haplotypes; π = 0.0066) (Figure 1; Figure S1; Tables S1 and S2). The most common mtDNA haplotype H11 was ubiquitous in Uganda/Tanzania and parts of Botswana/South Africa, H1 was common in Southern Africa, and H7 and H8 were unique to Asian lions. The autosomal nDNA sequences showed fairly distinct patterns of variation (Figure 1; Figure S1). Of the five *ADA* haplotypes, A2 was the most common and most-widely distributed. The other four haplotypes, which are derived and much less common, included one (A5) that was fixed in Asian lions. The three *TF* haplotypes were more widely and evenly distributed.

**Figure 2:**
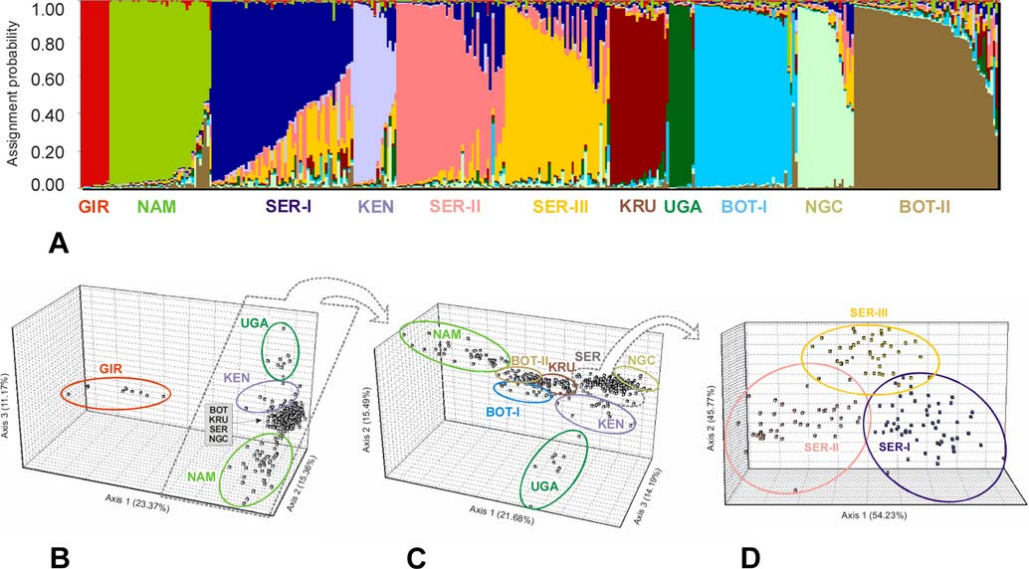
Population structure analyses in lions. (A) Bayesian population assignment test [22] of the 357 lions using 24 nDNA loci (*ADA*, *TF*, and 22 microsatellites) and mtDNA data, and considering *K* = 11 (11 populations). (B) Three-dimensional factorial correspondence analysis [23] (FCA) based on the 24 nDNA loci genotypes in the 357 lions. Axe 1, 2, and 3 represent 49.90% of the genetic variation observed. (C) FCA representation excluding the GIR lions. Axe 1, 2, and 3 represent 51.35% of the genetic variation observed. (D) FCA representation considering only the SER lions supportive of a three distinct population clusters subdivision (SER-I, SER-II, and SER-III).

Levels of population subdivision among lions were assessed using microsatellite and sequencing data. Eleven groups were identified using Bayesian analyses [22] and three-dimensional factorial correspondence analyses [23] (Figure 2; Table S3). Most clusters represented geographically circumscribed populations: Namibia (Nam), Kruger National Park (Kru), Ngorongoro Crater (Ngc), Kenya (Ken), Uganda (Uga), and Gir (Gir). Two distinct clusters were found in Botswana, Bot-I that included lions from southern Botswana and Kalahari (South Africa) (*F*_k_ = 0.24) and Bot-II found exclusively in northern Botswana (*F*_k_ = 0.18). Surprisingly, three distinct clusters were found in a single geographical locale (approximately 60×40 km square) in the large panmyctic population of the Serengeti National Park (Ser-I/Ser-II/Ser-III) (*F*_k_ = 0.18, 0.21, and 0.15, respectively).

Two captive lions from Angola (Ang), one from Zimbabwe (Zbw) and four Morocco Zoo Atlas lions (Atl; presently extinct from the wild) (Figure 1A) were included in the analyses to explore the relationship of lions from more isolated, endangered, or depleted areas. Ang and Zbw lions were assigned to Bot-II (*q* = 0.90 and 0.87; 90%CI: 0.47–1.00) and Kru (*q* = 0.85; 90%CI: 0.52–1.00) (Bayesian analyses [22]) populations, respectively, as expected based on their geographical proximity. However, these lions differed from Bot-II and Kru by up to 8 mtDNA mutations, sharing haplotypes with the Atl lions (H5 in Ang and H6 in Zbw) (Figure 1B and 1C). The Atl lions did not group in a unique cluster.

**Figure 3:**
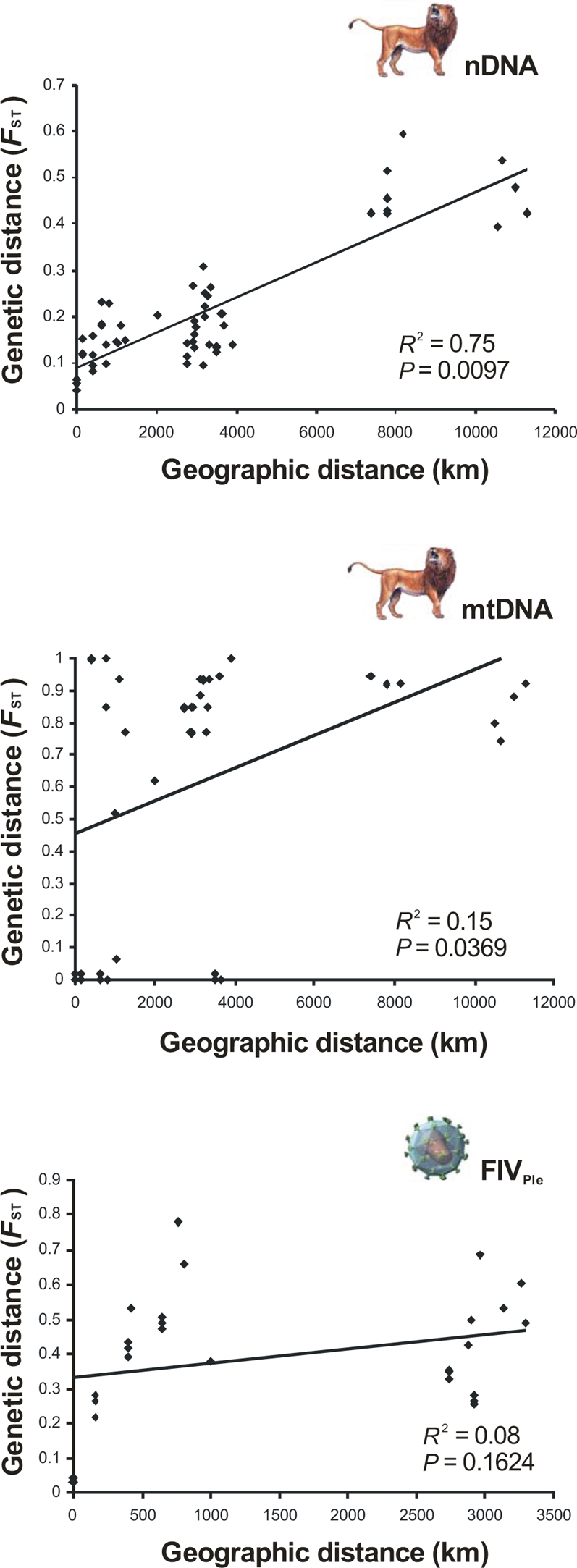
Genetic differentiation of host and viral genetic markers with geographic distance. Regression of lion pairwise *F*_ST_ (nDNA and mtDNA) and FIV_Ple_ (*pol*-RT) on geographic distance.

Both nDNA and mtDNA pairwise genetic distances among the 11 lion populations showed a significant relationship with geographic distance (*R* ^2^ = 0.75; Mantel's test, *P* = 0.0097; and *R* ^2^ = 0.15; Mantel's test, *P* = 0.0369; respectively) (Figure 3). The significant positive and monotonic correlation across all the scatterplot pairwise comparisons for the nDNA markers (bi-parental) was consistent with isolation-by-distance across the sampled region. However, the correlation between nDNA *F*_ST_ and geographic distance considerably decreased when the Asian Gir population was removed (*R* ^2^ = 0.19; Mantel's test, *P* = 0.0065) suggesting that caution should be taken in interpreting the pattern of isolation-by-distance in lions. We further compared linearized *F*_ST_ estimates [24] plotted both against the geographic distance (model assuming habitat to be arrayed in an infinite one-dimensional lattice) and the log geographic distance (model assuming an infinite two-dimensional lattice). The broad distribution of lions might suggest *a priori* that a two-dimensional isolation-by-distance model would provide the best fit for the nDNA data (*R* ^2^ = 0.25; Mantel's test, *P* = 0.0022), but instead the one-dimensional isolation-by-distance model performed better (*R* ^2^ = 0.71; Mantel's test, *P* = 0.0476) (Figure S2).

The pattern observed for the mtDNA (maternal) was more complex. While there was a significant relationship between mtDNA *F*_ST_ and geographic distance, there was an inconsistent pattern across broader geographic distances (Figure 3). This is partly due to the fixation or near fixation of haplotype H11 in six populations and the fixation of a very divergent haplotype H4 in Ken population (Figure 1B and 1C). The removal of the Ken population considerably increased the correlation between mtDNA *F*_ST_ and geographic distance (*R* ^2^ = 0.27; Mantel's test, *P* = 0.0035). Thus, the null hypothesis of regional equilibrium for mtDNA across the entire sampled region is rejected despite the possibility that isolation-by-distance may occur regionally.

**Figure 4:**
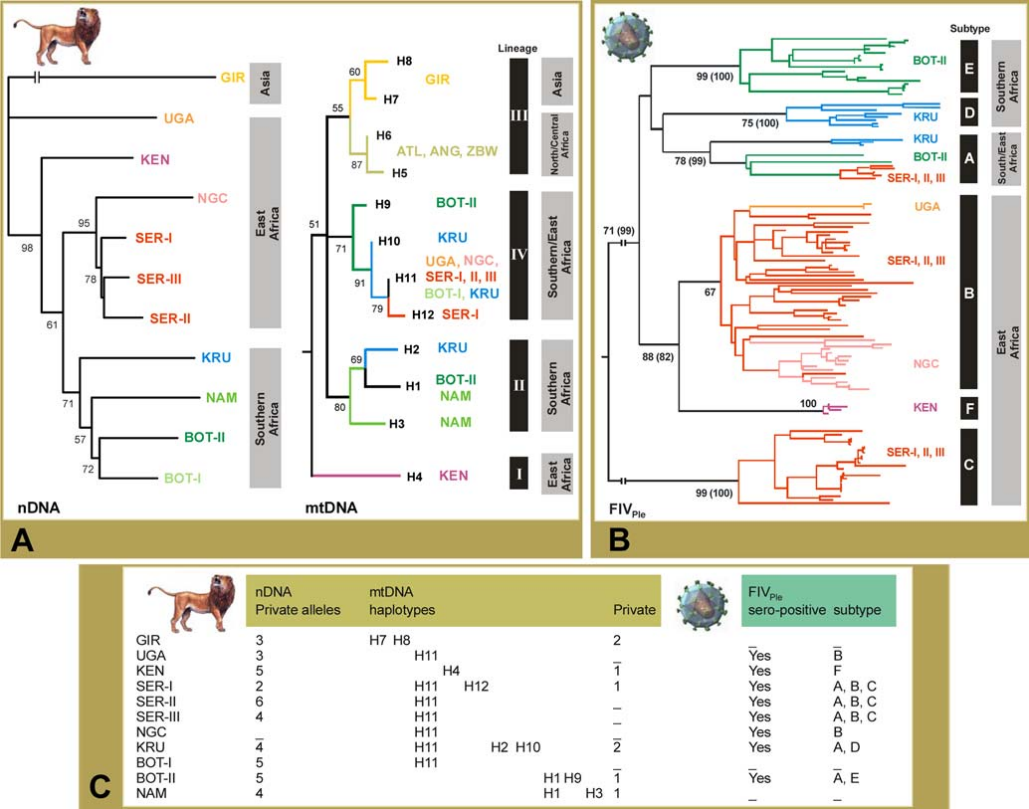
Evolutionary relationships of the host and viral genetic markers among lion populations. (A) Unrooted neighbour-joining (NJ) tree from nDNA genotypes of 24 loci (*ADA*, *TF*, and 22 microsatellites) in the 11 lion populations (left), and rooted NJ tree for the distinct mtDNA (*12S*–*16S*, 1,882 bp) haplotypes in lion (right). The distinct mtDNA lineages were labelled I to IV. Bootstrap support (BPS) values >50 are indicated. (B) NJ tree of the 301 bp FIV_Ple_ *pol*-RT sequences. The distinct FIV_Ple_ subtypes were labelled A to F. BPS values are placed at each branchpoint and in parenthesis are the BPS values obtained for a tree established with 520 bp of FIV_Ple_ *pol*-RT sequence for a representative subset of individuals. (C) Distinctiveness of host and viral molecular genetics in lion populations.

These contrasting nDNA and mtDNA results may be indicative of differences in dispersal patterns between males and females, which would be consistent with evidence that females are more phylopatric than males. Alternatively, selection for matrilineally transmitted traits upon which neutral mtDNA alleles hitchhike is possible, given the low values of nucleotide diversity of the mtDNA (π = 0.0066). A similar process has been suggested in whales (π = 0.0007) [25] and African savannah elephants (π = 0.0200) [26], where both species have female phylopatry and like lions, a matriarchal social structure. However, genetic drift tends to overwhelm selection in small isolated populations, predominantly affecting haploid elements due to its lower effective population size (Table 1). Therefore, we suggest that the contrasting results obtained for nDNA and mtDNA are more likely further evidence that lion populations underwent severe bottlenecks. The highly structured lion matrilines comprise four monophyletic mtDNA haplo-groups (Figure 4A; Figure S3). Lineage I consisted of a divergent haplotype H4 from Ken, lineage II was observed in most Southern Africa populations, lineage III was widely distributed from Central and Northern Africa to Asia, and lineage IV occurred in Southern and Eastern Africa.

### Population Structure of FIV_Ple_

Seroprevalence studies indicate that FIV_Ple_ is endemic in eight of the 11 populations but absent from the Asian Gir lions in India and in Namibia and southern Botswana/Kalahari regions (Nam/Bot-I) in Southwest Africa (Figure 1B). Phylogenetic analysis of the conserved *pol*-RT region in 117 FIV-infected lions depicted monophyletic lineages [15],[18] that affirm six distinct subtypes (A–F) that are distributed geographically in three distinct patterns (Figure 4B; Figure S4). First, multiple subtypes may circulate within the same population as exemplified by subtypes A, B and C all ubiquitous within the three Serengeti lion populations (Ser-I, Ser-II and Ser-III) and subtypes A and E within lions of Botswana (Bot-II) (Figure 4B and 4C and Figure S4). Second, unique FIV_Ple_ subtypes may be restricted to one location as subtype F in Kenya, subtype E in Botswana (Bot-II), subtype C in Serengeti, and subtype D in Krugar Park (Figure 4B and 4C and Figure S4). Third, intra-subtype strains cluster geographically, as shown by distinct clades within subtype A that were restricted to lions within Krugar Park, Botswana and Serengeti and within subtype B that corresponded to Uganda, Serengeti and Ngorongoro Crater lions (Figure 4B and 4C and Figure S4).

Not unexpectedly, FIV_Ple_ pairwise genetic distances, represented as population *F*_ST_ among the eight lion FIV-infected populations, were not significantly correlated with geographic distance (*R* ^2^ = 0.08; Mantel's test, *P* = 0.165) (Figure 3), and affirms that patterns of viral dissemination do not conform to a strict isolation-by-distance model. Rather, the two distinct clusters observed (Figure 3) reflect the complex distribution of FIV_Ple_ among African lions. Indeed, despite the low geographic distance within East-African lion populations, the FIV_Ple_ genetic divergence showed a broader range in *F*_ST_ (0.03 to 0.79 for most of first cluster; Figure 3). By contrast, approximately half of the range in *F*_ST_ (0.26 to 0.69 for the second cluster; Figure 3) was observed among East and Southern Africa in spite of its large geographic separation. In contrast with the patterns observed in lions, linearized *F*_ST_ estimates [24] for FIV_Ple_ were better correlated with log geographic distance (two-dimensional lattice model) (*R* ^2^ = 0.15) than with geographic distance (one-dimensional model) (*R* ^2^ = 0.02), although in both cases the Mantel's test was not significant (*P*>0.2474) (Figure S2).

### Natural History of Lions as Inferred from Lion and FIV_Ple_ Markers

The mtDNA coalescence dating suggested that the East African lineage I (Ken haplotype H4) had an old origin of ∼324,000 years (95% CI: 145,000–502,000). Extant East African populations (Ken/Ngc/Ser-I/Ser-II/Ser-III) also showed a slightly significant higher nDNA allelic richness and genetic diversity (Table S4) relative to populations to the south (Kru/Nam/Bot-I/Bot-II) and north (Uga/Gir) (*A* = 2.43, 2.39, and 1.62, *P* = 0.021; *H*_O_ = 0.64, 0.62, and 0.34, *P* = 0.019; respectively). Moreover, the FIV_Ple_ subtype diversity was higher in East African clades (exhibiting four out of the six known viral-strains), including the most divergent FIV_Ple_ subtype C (Figure 4B and 4C). These genetic data from lions and FIV_Ple_ is consistent with the older origin of extant East African lions, which is further supported by the oldest lion fossils discovered in East Africa [1].

Relative to East Africa, Southern lions have a slightly more recent mtDNA coalescence. Lineage II, found in Nam, Bot-II and Kru has an estimated coalescence of 169,000 years (95% CI: 34,000–304,000) and the more widespread lineage IV found in the Southern populations of Bot-I, Bot-II and Kru as well as the Eastern populations of Ser (I, II, and III), Ngc and Uga, coalesces ∼101,000 years ago (95% CI: 11,000–191,000). However, the similar levels of nDNA genetic diversity, the occurrence of an exclusively Southern mtDNA lineage II and highly divergent FIV_Ple_ subtypes, FIV_Ple_ subtype D found only in Kru and subtype E exclusive to Bot-II, suggests that both East and Southern Africa were important refugia for lions during the Pleistocene. Therefore, the co-occurrence of divergent mtDNA haplotypes (6 to 10 mutations; Figure 1B and 1C) in southern populations may be the consequence of further isolation within refugia during colder climatic periods. Contemporary fragmentation of lion populations could further explain the results of nested-clade phylogeographical analysis (NCPA [27]) (Figure S5), which inferred restricted gene flow with isolation-by-distance between mtDNA haplotypes H9 (Bot-II) and H10 (Kru) (χ^2^ = 10.00, *P* = 0.0200), between haplotypes H1 (Bot-II/Nam) and H2 (Kru) (χ^2^ = 71.00, *P*≤0.0001), and between haplotypes H9–H10 (Bot-II/Kru) and haplotypes H11–H12 (Bot-I/Kru/Ser/Ngc/Uga) (χ^2^ = 187.83, *P*≤0.0001).

Further isolation within refugia (sub-refugia) may also have occurred in East Africa. This is suggested by the distinctive mtDNA haplotype H4 and the unique FIV_Ple_ subtype F found in the Kenya population, which may have resulted from reduced gene flow across the Rift valley, a scenario that has been suggested for several bovid and carnivore populations (see [28] and references therein).

The best example of concordance between host genome markers and viral transmission patterns is observed in the Serengeti National Park in Tanzania. Our previous findings described markedly high levels of FIV_Ple_ subtype A, B and C circulating within the Serengeti lion population to such an extent that 43% of the lions sampled were multiply-infected with two or three subtypes [15],[18] and were hypothesized to represent recent admixture of three formerly separated populations. Such result is confirmed here by lion genomic markers (Figure 2). Further, although lions within the Serengeti can be assigned to one of three populations (Ser-I, Ser-II or Ser-III) by host genomic markers, FIV_Ple_ subtypes are distributed ubiquitously in all three, characteristic of rapid horizontal retroviral transmission subsequent to host population admixture. The possible isolating mechanism remains to be elucidated as there is no apparent barrier to gene flow in this ecosystem.

### Genomic Signatures Left by Migration

Based on patterns of genetic diversity and phylogenetic analysis of lion nDNA/mtDNA and FIV_Ple_ markers, we propose a scenario of a period of refugia/isolation in the Late Pleistocene followed by two major lion expansions across Africa and Asia. The first expansion, supported by the mtDNA NCPA [27] (χ^2^ = 690.00, *P*≤0.0001; Figure S5), was a long-distance colonization of mtDNA lineage-III (Gir/Atl/Ang/Zbw) around 118,000 years ago (95% CI: 28,000–208,000), with subsequent fragmentation of haplotypes H5–H6 into Central and North Africa and haplotypes H7–H8 into West Asia (M1- Figure 1A). Support for this initial expansion is also found in nDNA. The *ADA* haplotype A5 fixed in Gir in also present in Ken, Ser-II, and Ser-III, suggesting that lions likely colonized West Asia from the East Africa refugia (Figure 1B). Such an expansion may have been favored by the start of a warmer and less arid period in Africa 130,000–70,000 years ago [29]. This “out-of-Africa event” would have occurred much later than the initial lion expansion through Eurasia based on fossils (∼500,000 years ago) [3]. It is likely that multiple lion expansions occurred in the Pleistocene, as occurred with humans [21].

A second, more recent lion expansion probably occurred at the Pleistocene/Holocene transition, this one from Southern Africa toward East Africa (M2- Figure 1A, Figure 3). This is reflected in the mtDNA linage IV, where haplotypes present in Southern lions are basal (older) to those found in the East. Overall, mtDNA population nucleotide diversity decreases from Southern to East Africa (Figure 1B and 1C), a finding supported by pairwise mismatch analysis [30] (raggedness, *r* = 0.086; *P*<0.001). The fixation of mtDNA haplotype H11 in Bot-I (otherwise fixed only in East Africa populations) suggests that the colonizing lions expanded northwards from the Kalahari Desert, which included bush, woodland and savannah habitats during the climatic fluctuations of the Pleistocene [31]. This expansion would have occurred relatively recently as the single rare tip mtDNA haplotype H12, found only in Ser-I, is derived from the interior widespread haplotype H11 (∼14,000–7,000 years; given one mtDNA substitution every 7,000 years; Table 1). This expansion is also supported by FIV_Ple_ subtype A where haplotypes present in Southern lions (Kru and Bot-I) are basal to those found in the East (Ser-I, Ser-II and Ser-III) and a decrease of nucleotide-diversity of this FIV_Ple_ subtype is observed from Southern (π = 0.15) to Eastern Africa (π = 0.03) (Figure 3B). Interestingly, a similar northward colonization process from Southern Africa has been suggested for some of the lion preys, namely the impala, greater kudu, and wildebeest [32],[33].

### Utility of Population Genomic Datasets

If we had restricted our inferences to mtDNA, we might have concluded that East African lion populations, which are fixed or nearly fixed for haplotype H11, went extinct during the Pleistocene/Holocene transition (similar to the well known mega-fauna extinctions of the Late Pleistocene [34]) and were then colonized by Southern populations. However, our population genomics data better fit a scenario of lion population expansion and interbreeding rather than simple replacement. First, genetic diversity and allelic richness at nDNA are slightly higher in East Africa populations relatively to those in Southern Africa. This is contrary to the expected pattern of population expansion in which there is usually a progressive decline in genetic diversity and allelic richness. Second, Ser lions carry two diverse FIV_Ple_ subtypes found only in East Africa (B–C), and not only FIV_Ple_ subtype A, which was presumably introduced in East Africa coincidently with the mtDNA expansion event northwards from South. Third, the East African FIV_Ple_ subtype B found in Uga/Ser-I/Ser-II/Ser-III/Ngc showed evidence of a population expansion (raggedness, *r* = 0.004; *P*<0.01; *F*_s_ = −20.37; *P*<0.00001) and the highest nucleotide diversity observed within FIV_Ple_ subtypes (π = 0.09). Four, the FIV_Ple_ subtype diversity is higher in East African clades (four out of the six viral strains).

The utility of FIV_Ple_ *pol*-RT as a marker of lion population structure and natural history is that it can be informative on a contemporaneous time scale, though it may be less useful at capturing more ancient demographic events. The extreme divergence among FIV_Ple_ subtypes, considered with high sero-prevalence in eight of the 11 lion populations, and combined with patterns of geographic concordance, support the hypothesis that FIV_Ple_ is not a recent emergence within modern lions [35]. Populations that harbor one private FIV_Ple_ subtype such Ken (subtype F), Bot-II (subtype E), and Kru (subtype D) must have been sufficiently isolated for enough time for the virus to evolve into unique subtypes, a result corroborated by the high nDNA and mtDNA genetic structure present in these lion populations (Figure 4). Thus, it is possible that the initial emergence of FIV_Ple_ pre-dates the Late-Pleistocene expansions of contemporary lion populations [36], but present day distributions are more useful indicators of very recent host population dynamics, a result also observed with FIV_Pco_ in a panmictic population of pumas in western North America [19].

### Conservation Implications

Accurate interpretation of past and contemporary population demographic scenarios is a primary goal for the effective conservation of endangered species. In this study, we found substantial population subdivision, reduced gene flow, and large differences in FIV_Ple_ sequence and sero-prevalence among lion populations, as well as evidence of historic secondary contact between populations (Figure 3C; Table S4 to S9). The very low population level of mtDNA nucleotide diversity, the number of haplotypes private to a single population (Figure 1), and probably also the lack of *SRY* genetic variation across all male lions (haplotype S1, *n* = 183) suggests that lion numbers diminished considerably following the Late Pleistocene. The last century reduction in lion distribution further eroded its genomic diversity, and microsatellite variation suggested recent population bottlenecks in seven out of the 11 populations (standardized differences test, *P*<0.05; Table S5) [37].

Although we did not explicitly try to address the adequacy of lion subspecies designations (currently only one African subspecies is widely recognized) [38],[39], we provided strong evidence that there is no evidence of substantial genetic exchange of matrilines among existing populations as the AMOVA [40] within-population component was uniformly high in all distinct subdivision scenarios (Φ_ST_≈0.920; *P*<0.0001; three-six groups; Table S6). Similarly, significant population structure was detected from nDNA (*F*_ST_ = 0.18), with low levels of admixture evident from Bayesian analysis [22] (α = 0.033). Therefore, employing a bottom-up perspective that prioritizes populations, rather than large-scale units (e.g. all African lions), might preserve and maintain lion diversity and evolutionary processes most efficiently [41].

## Material and Methods

### Study Site, Sampling, and Molecular Genetic Analyses of Lions

A total of 357 individuals were obtained across most of the lion range in Africa and Asia (Figure 1A; Table S1). Genetic variation among lion specimens was assessed using maternal (*12S* and *16S* genes), paternal (*SRY* gene) and bi-parental autosomal (22 microsatelite loci, and the *ADA* and *TF* genes) markers (GenBank accession numbers: FJ151632–FJ151652). Analyses of mtDNA in *Panthera* species are complicated by the presence of a 12.5 kb mtDNA integration into chromosome F2 [42]. Accordingly, mtDNA specific primers were designed for the *12S* and *16S* genes (Table S2) and we used long-range PCR amplification. We designed primers to amplify segments of the *ADA* (exon 10 and intron 10) and the *TF* (intron 3) genes (Table S2), two of the most variable protein loci in lion populations [5], localized on the domestic cat *Felis catus* chromosome A3p and C2q, respectively. The Y-chromosome *SRY*-3′UTR gene was also amplified [43].

PCR products were amplified from 50 ng of genomic DNA in a 25 µL reaction system containing 1.5 mM MgCl_2_, 1.0 mM dNTPs, 0.25 units of AmpliTaq Gold DNA polymerase (Applied Biosystems), and 1× PCR buffer II; the amplification protocol was: denaturation 10 min at 95°C, a touch-down cycle of 95°C for 30 s, 52°C for 60 s decreased by 1°C in the next cycle for 10 cycles, 72°C for 120 s, then 35 amplification cycles of 95°C for 30 s, 52°C for 60 s, and 72°C for 120 s, followed by an extension of 10 min at 72°C. PCR products were sequenced on an ABI 377. Sequences were aligned and cleaned using SEQUENCHER (Gene Codes).

Twenty two polymorphic microsatellite loci (20 dinucleotide repeats: FCA006, FCA008, FCA014, FCA069, FCA077, FCA085, FCA091, FCA098, FCA105, FCA126, FCA129, FCA139, FCA205, FCA208, FCA211, FCA224, FCA229, FCA230, FCA247, and FCA281; and two tetranucleotide repeats: FCA391 and FCA441) were amplified [44]. Microsatellites were scored in an ABI 377 and analyzed using Genescan 2.1 and Genotyper 2.5. These loci are located on 11 of the 19 *F. catus* chromosomes, occurring in different linkage groups or at least 12 centimorgans apart [44],[45].

### Sero-Prevalence and Molecular Genetics of FIV_Ple_

Western blots using domestic cat and lion FIV as antigen were performed as previously described [46],[47]. The supernatant from virus-infected cells was centrifuged at 200 g for 10 min at 5°C. The resultant supernatant was centrifuged at 150,000 g at 4°C for 2 hours. Pelleted viral proteins were resuspended in 1/20^th^ of the original volume and total protein content was assayed using the Biorad Protein Assay. Twenty mg of viral protein were run on 4–20% Tris-Glycine gels and transferred to PDVF membranes (BioRad). Membrane strips were exposed 2–12 h to a 1∶25 or 1∶200 dilution of serum or plasma. After washing, samples were labeled with goat anti-cat HRP or phosphate conjugated antibody (KPL laboratories) at a 1∶2000 dilution, washed, and incubated in ECL Western Blotting detection reagents (Amersham Biosciences) for 2 min, then exposed to Lumi-Film Chemiluminescent Detection Film (Boehringer Mannheim) or incubated in BCIP/NBP phosphatase substrate (KPL laboratories) for 15 min [46]–[48]. Results were visualized and scored manually based on the presence and intensity of antibody binding to the *p24 gag* capsid protein.

Nested PCR amplification of partial FIV_Ple_ *pol*-RT was performed [18],[46]. Briefly, first round PCR reactions used 100 ng of genomic DNA, 2.5 mM MgCl_2_ and an annealing temperature of 52°C. Second round PCR reactions used identical conditions with 1–5 µl of first-round product as template. All PCR products were sequenced as described above for lion genetic analyses (GenBank accession numbers: AY549217–AY552683; AY878208–AY878235; FJ225347–FJ225382).

### Statistical Analyses

We used the Genetix 4.02 [49], Genepop 3.3 [50], Microsat [51], and DnaSP 4.10 [52] to calculate the following descriptive statistics: (i) percentage of polymorphic loci (*P*_95_), number of alleles per locus (*A*), observed and expected heterozygosity (*H*_E_ and *H*_O_), and number of unique alleles (*A*_U_); (ii) assess deviations from HWE; (iii) estimate the coefficient of differentiation (*F*_ST_), and (iv) nucleotide (π) and haplotype (*h*) diversity.

We tested the hypothesis that all loci are evolving under neutrality for both the lion and the FIV_Ple_ data. For frequency data, we used the method described by Beaumont and Nichols [53] and implemented in Fdist (http://www.rubic.rdg.ac.uk/˜mab/software.html). The *F*_ST_ values estimated from microsatellite loci plotted against heterozygosity showed that all values fall within the expected 95% confidence limit and consequently no outlier locus were identified. For sequence data (lion nDNA/mtDNA and FIV_Ple_ *pol*-RT), we ruled out any significant evidence for genetic hitchhiking and background selection by assessing Fu and Li's D* and F* tests [54] and Fu's *F*_S_ statistics [55].

A Bayesian clustering method implemented in the program Structure [22] was used to infer number of populations and assign individual lions to populations based on multilocus genotype (microsatellites) and sequence data (*ADA*, *TF*, and mtDNA genes) and without incorporating sample origin. For haploid mtDNA data, each observed haplotype was coded with a unique integer (e.g. 100, 110) for the first allele and missing data for the second (Structure [22] analyses with or without the mtDNA data were essentially identical). For *K* population clusters, the program estimates the probability of the data, Pr(*X|K*), and the probability of individual membership in each cluster using a Markov chain Monte Carlo method under the assumption of Hardy-Weinberg equilibrium (HWE) within each cluster. Initial testing of the HWE in each of the populations defined by the geographic origin of sampling revealed no significant deviation from HW expectations with the exception of Ser and Bot population (later subdivided by Structure [22] in 3 and 2 clusters, respectively; such deviations from HW expectations were interpreted as evidence of further population structuring). We conducted six independent runs with *K* = 1–20 to guide an empirical estimate of the number of identifiable populations, assuming an admixture model with correlated allele frequencies and with burn-in and replication values set at 30,000 and 10^6^, respectively. Structure also estimates allele frequencies of a hypothetical ancestral population and an alpha value that measures admixed individuals in the data set. The assignment of admixed individuals to populations using Structure [22] has been considered in subsequent population analyses. For each population cluster *k*, the program estimates *F*_k_, a quantity analogous to Wright's *F*_ST_, but describing the degree of genetic differentiation of population *k* from the ancestral population.

Patterns of gene flow and divergence among populations were described using a variety of tests. First, to visualize subtle relationships among individual autosomal genotypes, three-dimensional factorial correspondence analyses [23] (FCA) were performed in Genetix [49], which graphically projects the individuals on the factor space defined by the similarity of their allelic states. Second, neighbor-joining (NJ) analyses implementing the Cavalli-Sforza & Edwards' chord genetic distance [56] (*D*_CE_) were estimated in Phylip 3.6 [57], and the tree topology support was assessed by 100 bootstraps. Third, the difference in average *H*_O_ and *A* was compared among population groups using a two-sided test in Fstat 2.9.3.2 [58], which allows to assess the significance of the statistic OS_x_ using 1,000 randomizations. Four, the equilibrium between drift and gene flow was tested using a regression of pairwise *F*_ST_ on geographic distance matrix among all populations for host nDNA(microsatellites)/mtDNA and FIV_Ple_ data. A Mantel test [59] was used to estimate the 95% upper probability for each matrix correlation. Assuming a stepping stone model of migration where gene flow is more likely between adjacent populations, one can reject the null hypothesis that populations in a region are at equilibrium if (1) there is a non-significant association between genetic and geographic distances, and/or (2) a scatterplot of the genetic and geographic distances fails to reveal a positive and monotonic relationship over all distance values of a region [60]. We also evaluated linearized *F*_ST_ [i.e. *F*_ST_/(1−*F*_ST_)] [24] among populations. We tested two competing models of isolation-by-distance, one assuming the habitat to be arrayed in an infinite one-dimensional lattice and another assuming an infinite two-dimensional lattice. Both models showed that genetic differentiation increased with raw and log-transformed Euclidean distances, respectively [24]. We determined the confidence interval value of the slope of the regression for the nDNA data using a non parametric ABC bootstrap [61] in Genepop 4.0 [62].

The demographic history of populations was compared using a variety of estimators based on the coalescence theory. First, signatures of old demographic population expansion were investigated for mtDNA and FIV_Ple_ *pol*-RT haplotypes using pairwise mismatch distributions [63] in DnaSP [52]. The goodness-of-fit of the observed data to a simulated model of expansion was tested with the raggedness (*r*) index [64].

Second, the occurrence of recent bottlenecks was evaluated for microsatellite data using the method of Cornuet & Luikart [37] in Bottleneck [65] and using 10,000 iterations. This approach, which exploits the fact that rare alleles are generally lost first through genetic drift after reduction in population size, employs the standardize differences test, which is the most appropriate and powerful when using 20 or more polymorphic loci [37]. Tests were carried out using the stepwise mutation model (SMM), which is a conservative mutation model for the detection of bottleneck signatures with microsatellites [66].

Third, to discriminate between recurrent gene flow and historical events we used the nested-clade phylogeographical analysis [27],[67] (NCPA) for the mtDNA data. When the null-hypothesis of no correlation between genealogy and geography is rejected, biological inferences are drawn using a priori criteria. The NCPA started with the estimation of a 95% statistical parsimony [68] mtDNA network in Tcs 1.20 [69]. Tree ambiguities were further resolved using a coalescence criteria [70]. The network was converted into a series of nested branches (clades) [71],[72], which were then tested against their geographical locations through a permutational contingency analysis in GeoDis 2.2 [73]. The inferences obtained were also corroborated with the automated implementation of the NCPA in ANeCA [74]. To address potential weaknesses in some aspects of the NCPA analysis [75],[76], we further validated the NCPA inferences with independent methods for detecting restricted gene-flow/isolation-by-distance (using matrix correlation of pairwise *F*_ST_ and geographic distance) and population expansion (using pairwise mismatch distributions).

Four, to test the significance of the total mtDNA genetic variance, we conducted hierarchical analyses of molecular variance [40] (AMOVA) using Arlequin 2.0 [77]. Total genetic variation was partitioned to compare the amount of difference among population groups, among populations within each groups, and within populations.

Phylogenetic relationships among mtDNA and FIV_Ple_ *pol*-RT sequences were assessed using Minimum evolution (ME), Maximum parsimony (MP), and Maximum likelihood (ML) approaches implemented in Paup [78]. The ME analysis for mtDNA consisted of NJ trees constructed from Kimura two-parameter distances followed by a branch-swapping procedure and for FIV_Ple_ data employed the same parameter estimates as were used in the ML analysis. The MP analysis was conducted using a heuristic search, with random additions of taxa and tree-bisection-reconnection branch swapping. The ML analysis was done after selecting the best evolutionary model fitting the data using Modeltest 3.7 [79]. Tree topologies reliability was assessed by 100 bootstraps. For the FIV_Ple_ data, the reliability of the tree topology was further assessed through additional analyses using 520 bp of FIV_Ple_ *pol*-RT sequences in a representative subset of individuals.

The time to the most recent common ancestor (TMRCA) for the *ADA* and *TF* haplotypes was estimated following Takahata et al. [80], where we calculate the ratio of the average nucleotide differences within the lion sample to one-half the average nucleotide difference between leopards (*P. pardus*) and lions and multiplying the ratio by an estimate of the divergence time between lions and leopards (2 million years based on undisputed lion fossils in Africa) [81],[82]. The mtDNA TMRCA was estimated with a linearized tree method in Lintree [83] and using the equation H = 2μT, where H was the branch height (correlated to the average pairwise distance among haplotypes), μ the substitution rate, and T the divergence time. Leopard and snow leopard (*P. uncia*) sequences were used as outgroups. Inference of the TMRCA for microsatellite loci followed Driscoll et al. [6] where the estimate of microsatellite variance in average allele repeat-size was used as a surrogate for evolutionary time based on the rate of allele range reconstitution subsequent to a severe founder effect. Microsatellite allele variance has been shown to be a reliable estimator for microsatellite evolution and demographic inference in felid species [6].

## Supporting Information

Figure S1 Genetic variation of *12S–16S* (mtDNA) and ADA and TF (nDNA) genes in lions. (A) Haplotypes and variable sites for the *12S–16S* mtDNA region surveyed in lions (total length 1,882 bp). Position 1 corresponds to position 1441 of the domestic cat (*Felis catus*) mtDNA genome (GenBank U20753). The “-” represents a gap and “.” matches the nucleotide in the first sequence. Shading indicates a fixed difference in the mtDNA lineage. (B) Haplotypes and variable sites for the *ADA* gene segment surveyed in lions (total length 427 bp). (C) Haplotypes and variable sites for the *TF* gene segment surveyed in lions (total length 427 bp).
(0.12 MB PDF)

Figure S2 Linearized genetic differentiation of host and viral genetic markers with geographic distance. Regression of linearized *F*_ST_ estimates [24] for lion (nDNA and mtDNA) and FIV_Ple_ (*pol*-RT) genetic data plotted both against the geographic distance (model assuming habitat to be arrayed in an infinite one-dimensional lattice; one-dimension isolation-by-distance [IBD]) and the log geographic distance (model assuming an infinite two-dimensional lattice; two-dimension isolation-by-distance) on geographic distance.
(0.13 MB PDF)

Figure S3 Phylogenetic relationships of the *12S–16S* mtDNA lion haplotypes. Neighbour-joining tree of the 1,882 bp *12S–16S* mtDNA sequences. Bootstrap values are placed at each branchpoint for the minimum evolution/maximum parsimony/maximum likelihood analyses, respectively (ME/MP/ML). Outgroups: Ppa – leopard, *Panthera pardus*; Pun – snow-leopard, *Panthera uncia*. The symbol (•) represents nodes with bootstrap support <50 or an inferred polytomy in the bootstrap 50% majority-rule consensus tree.
(0.10 MB PDF)

Figure S4 Phylogenetic relationships of the FIV_Ple_ *pol*-RT sequences. Neighbour-joining tree of the 301 bp FIV_Ple_ *pol*-RT sequences. The distinct FIV_Ple_ subtypes were labelled A to F. Bootstrap (BPS) values are placed at each branchpoint (ME/MP/ML) and in parenthesis are the BPS values obtained for a tree established with 520 bp of FIV_Ple_ *pol*-RT sequence for a representative subset of individuals.
(0.13 MB PDF)

Figure S5 Nested design and summary results of the nested clade phylogeographic analysis (NCPA) for lion mtDNA data. (A) Nested design of the mtDNA haplotype network used for the NCPA. (B) Summary results of the NCPA. RGF/IBD - Restricted gene flow/isolation by distance. LDC/FR – long distance colonization/fragmentation.
(0.10 MB PDF)

Table S1 List of the lion samples used in this study.
(0.11 MB PDF)

Table S2 Primers used to amplify the mtDNA (*12S–16S*) and nDNA (ADA and TF) portions surveyed in this study.
(0.06 MB PDF)

Table S3 Structure cluster assignment results of 357 lions based on nDNA (ADA, TF, and 22 microsatellites) and mtDNA markers. Burn-in and replication values set at 30,000 and 1,000,000, respectively.
(0.06 MB PDF)

Table S4 Gene diversity and frequency values in lion populations.
(0.07 MB PDF)

Table S5 Bottleneck analysis in lion populations using the standardized differences test and the stepwise mutation model (SMM).
(0.05 MB PDF)

Table S6 Results of the hierarchical AMOVA in lions for four different geographical scenarios.
(0.06 MB PDF)

Table S7 Lion population pairwise *F*_ST_ estimates. Below the diagonal mtDNA data (*12S–16S*) and above the diagonal microsatellite data (22 loci).
(0.06 MB PDF)

Table S8 Taxon specific unique nDNA alleles in lion populations (FCA-microsatellites and ADA locus).
(0.06 MB PDF)

Table S9 Summary statistics for FIV_Ple_ data.
(0.00 MB PDF)
