# Supplementary material for: The Evolutionary Dynamics of the Lion Panthera leo Revealed by Host and Viral Population Genomics
Source: PLoS Genet. 2008 Nov 7;4(11):e1000251. doi: 10.1371/journal.pgen.1000251 (PMC2572142; doi:10.1371/journal.pgen.1000251)
Supplement: Figure S1 — Genetic variation of 12S–16S (mtDNA) and ADA and TF (nDNA) genes in lions. (A) Haplotypes and variable sites for the 12S–16S mtDNA region surveyed in lions (total length 1,882 bp). Position 1 corresponds to position 1441 of the domestic cat (Felis catus) mtDNA genome (GenBank U20753). The “-” represents a gap and “.” matches the nucleotide in the first sequence. Shading indicates a fixed difference in the mtDNA lineage. (B) Haplotypes and variable sites for the ADA gene segment surveyed in lions (total length 427 bp). (C) Haplotypes and variable sites for the TF gene segment surveyed in lions (total length 427 bp). (0.12 MB PDF) [file pgen.1000251.s001.pdf]

12S-16S

Lineage	Haplotype			
			111	1111111
		2345556667	7888999001	2233666
		4661373893	9144622384	2428124
		2923111452	8201145924	0787096
II	H1	CCTCGGTTGC	TGTAAAACCA	ATATCCC
II	H2	....A.....	..C.-A....	.C...T.
II	H3	...TA.....	....-AG...	G....T.
I	H4	..C.A...AT	C...--G.T.	....TTT
III	H5	.T..A.C...	....--G...	.....TT
III	H6	.T..A.C...	.A..--G...	.....TT
III	H7	TT..A..C..	....--G..G	.....TT
III	H8	TT..A.C...	....--G..G	.....TT
IV	H9	....A..C..	...G--G...	..G..TT
IV	H10	....A..C..	....--G...	..GC.TT
IV	H11	....A..C..	....--GT..	..GC.TT
IV	H12	....AA.C..	....--GT..	..GC.TT

A

ADA

Haplotype		
	11133	33
	00300	23
	67918	17
A1	GGCCC	GG
A2	.....	A.
A3	.....	AT
A4	..G.T	A.
A5	--.T.	A.

B

TF

Haplotype	
	11
	225
	083
T1	ACT
T2	T.A
T3	TTA

C

Figure S1. Genetic variation of *12S-16S* (mtDNA) and *ADA* and *TF* (nDNA) genes in lions. (**A**) Haplotypes and variable sites for the *12S-16S* mtDNA region surveyed in lions (total length 1,882 bp). Position 1 corresponds to position 1441 of the domestic cat (*Felis catus*) mtDNA genome (GenBank U20753). The "-" represents a gap and "." matches the nucleotide in the first sequence. Shading indicates a fixed difference in the mtDNA lineage. (**B**) Haplotypes and variable sites for the *ADA* gene segment surveyed in lions (total length 427 bp). (**C**) Haplotypes and variable sites for the *TF* gene segment surveyed in lions (total length 427 bp).
