# Supplementary material for: The Evolutionary Dynamics of the Lion Panthera leo Revealed by Host and Viral Population Genomics
Source: PLoS Genet. 2008 Nov 7;4(11):e1000251. doi: 10.1371/journal.pgen.1000251 (PMC2572142; doi:10.1371/journal.pgen.1000251)
Supplement: Figure S2 — Linearized genetic differentiation of host and viral genetic markers with geographic distance. Regression of linearized F ST estimates [24] for lion (nDNA and mtDNA) and FIVPle (pol-RT) genetic data plotted both against the geographic distance (model assuming habitat to be arrayed in an infinite one-dimensional lattice; one-dimension isolation-by-distance [IBD]) and the log geographic distance (model assuming an infinite two-dimensional lattice; two-dimension isolation-by-distance) on geographic distance. (0.13 MB PDF) [file pgen.1000251.s002.pdf]

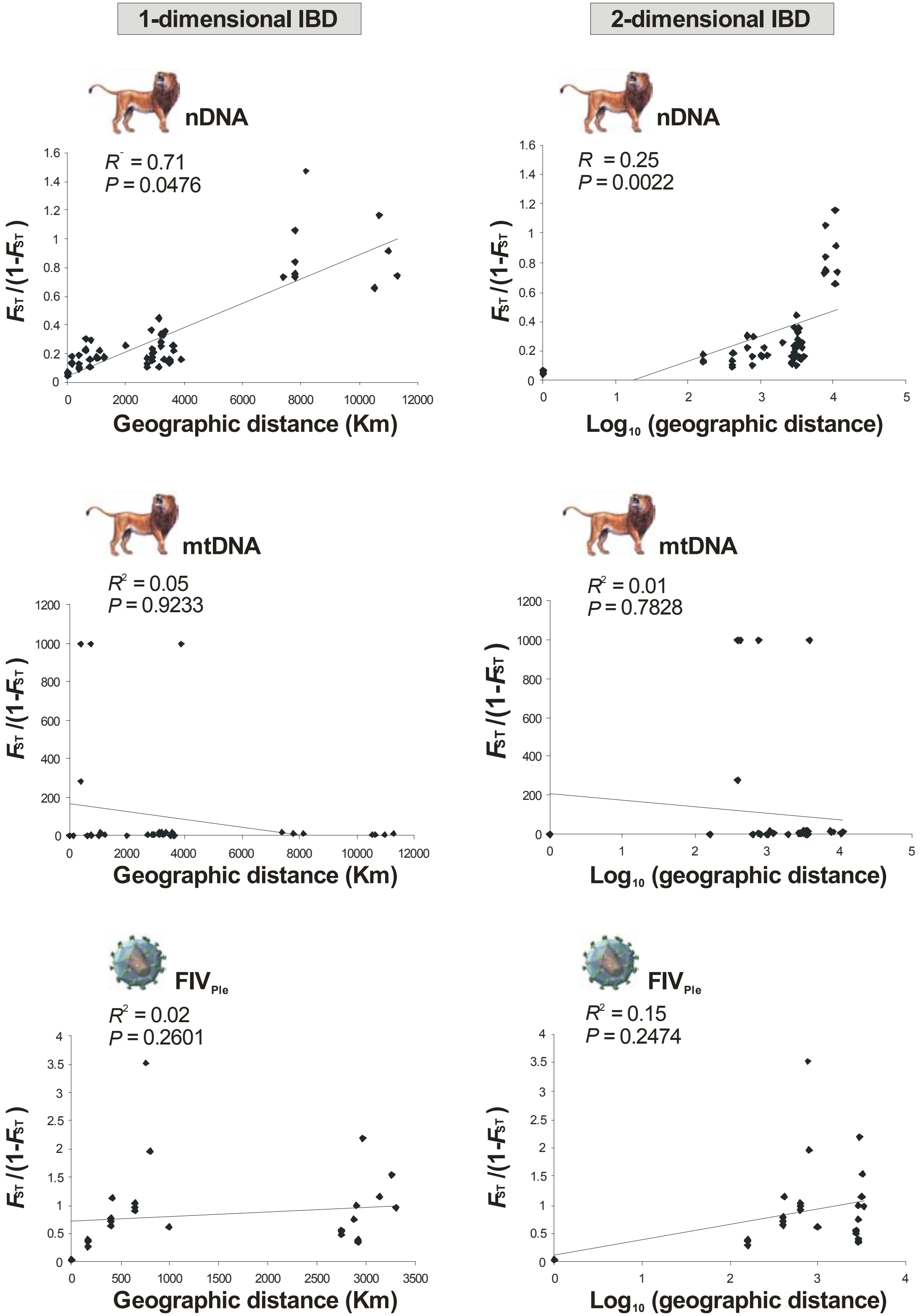

Figure S2. Linearized genetic differentiation of host and viral genetic markers with geographic distance. Regression of linearized F_{ST} estimates [24] for lion (nDNA and mtDNA) and FIV_{Ple} (*pol*-RT) genetic data plotted both against the geographic distance (model assuming habitat to be arrayed in an infinite one-dimensional lattice; one-dimension isolation-by-distance [IBD]) and the log geographic distance (model assuming an infinite two-dimensional lattice; two-dimension isolation-by-distance) on geographic distance.
