# Supplementary material for: The Evolutionary Dynamics of the Lion Panthera leo Revealed by Host and Viral Population Genomics
Source: PLoS Genet. 2008 Nov 7;4(11):e1000251. doi: 10.1371/journal.pgen.1000251 (PMC2572142; doi:10.1371/journal.pgen.1000251)
Supplement: Figure S3 — Phylogenetic relationships of the 12S–16S mtDNA lion haplotypes. Neighbour-joining tree of the 1,882 bp 12S–16S mtDNA sequences. Bootstrap values are placed at each branchpoint for the minimum evolution/maximum parsimony/maximum likelihood analyses, respectively (ME/MP/ML). Outgroups: Ppa – leopard, Panthera pardus; Pun – snow-leopard, Panthera uncia. The symbol (•) represents nodes with bootstrap support <50 or an inferred polytomy in the bootstrap 50% majority-rule consensus tree. (0.10 MB PDF) [file pgen.1000251.s003.pdf]

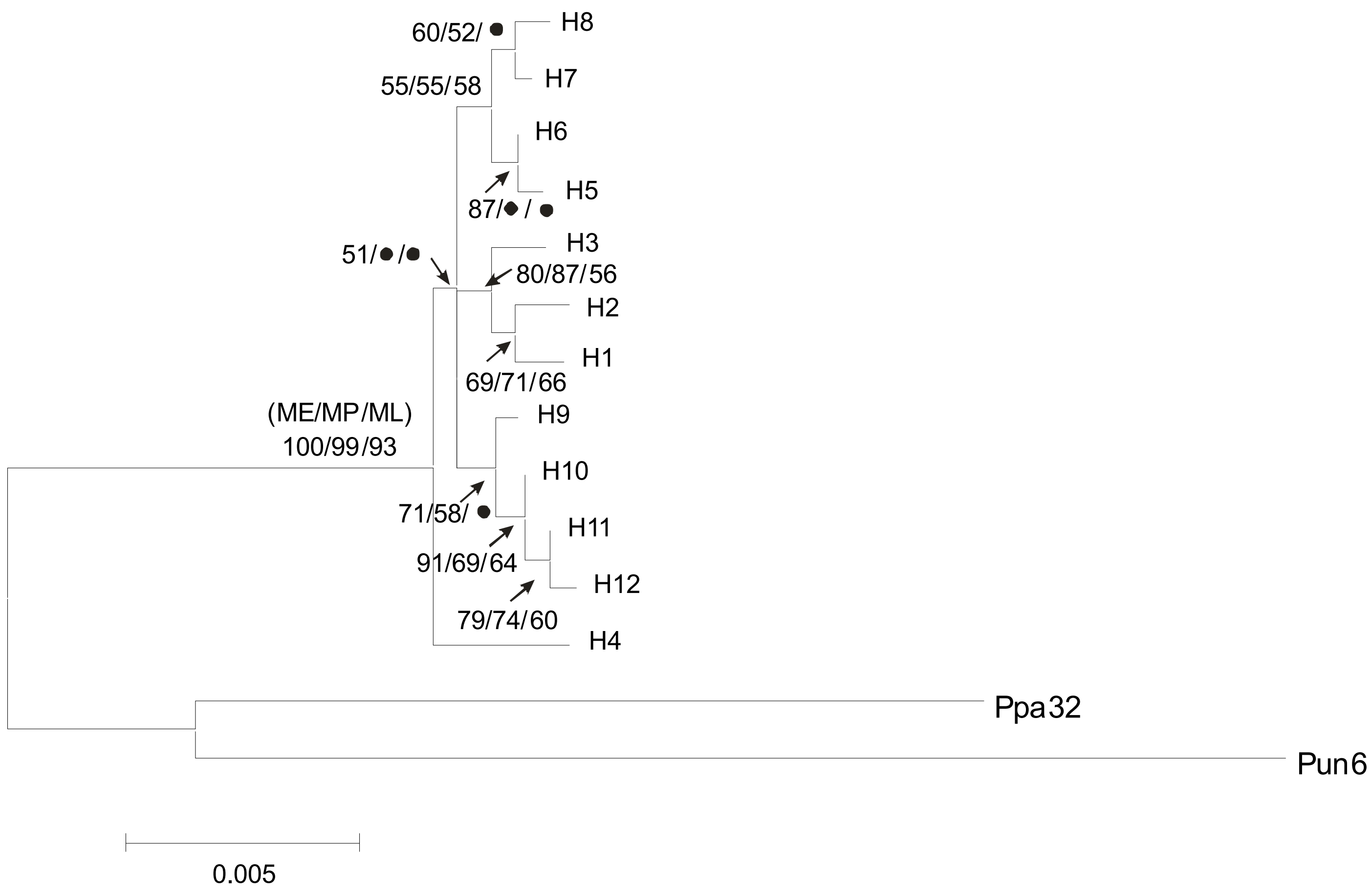

Figure S3. Phylogenetic relationships of the *12S-16S* mtDNA lion haplotypes. Neighbour-joining tree of the 1,882 bp *12S-16S* mtDNA sequences. Bootstrap values are placed at each branchpoint for the minimum evolution/maximum parsimony/maximum likelihood analyses, respectively (ME/MP/ML). Outgroups: Ppa – leopard, *Panthera pardus*; Pun – snow-leopard, *Panthera uncia*. The symbol (●) represents nodes with bootstrap support < 50 or an inferred polytomy in the bootstrap 50% majority-rule consensus tree.
