# Supplementary material for: The Evolutionary Dynamics of the Lion Panthera leo Revealed by Host and Viral Population Genomics
Source: PLoS Genet. 2008 Nov 7;4(11):e1000251. doi: 10.1371/journal.pgen.1000251 (PMC2572142; doi:10.1371/journal.pgen.1000251)
Supplement: Figure S4 — Phylogenetic relationships of the FIVPle pol-RT sequences. Neighbour-joining tree of the 301 bp FIVPle pol-RT sequences. The distinct FIVPle subtypes were labelled A to F. Bootstrap (BPS) values are placed at each branchpoint (ME/MP/ML) and in parenthesis are the BPS values obtained for a tree established with 520 bp of FIVPle pol-RT sequence for a representative subset of individuals. (0.13 MB PDF) [file pgen.1000251.s004.pdf]

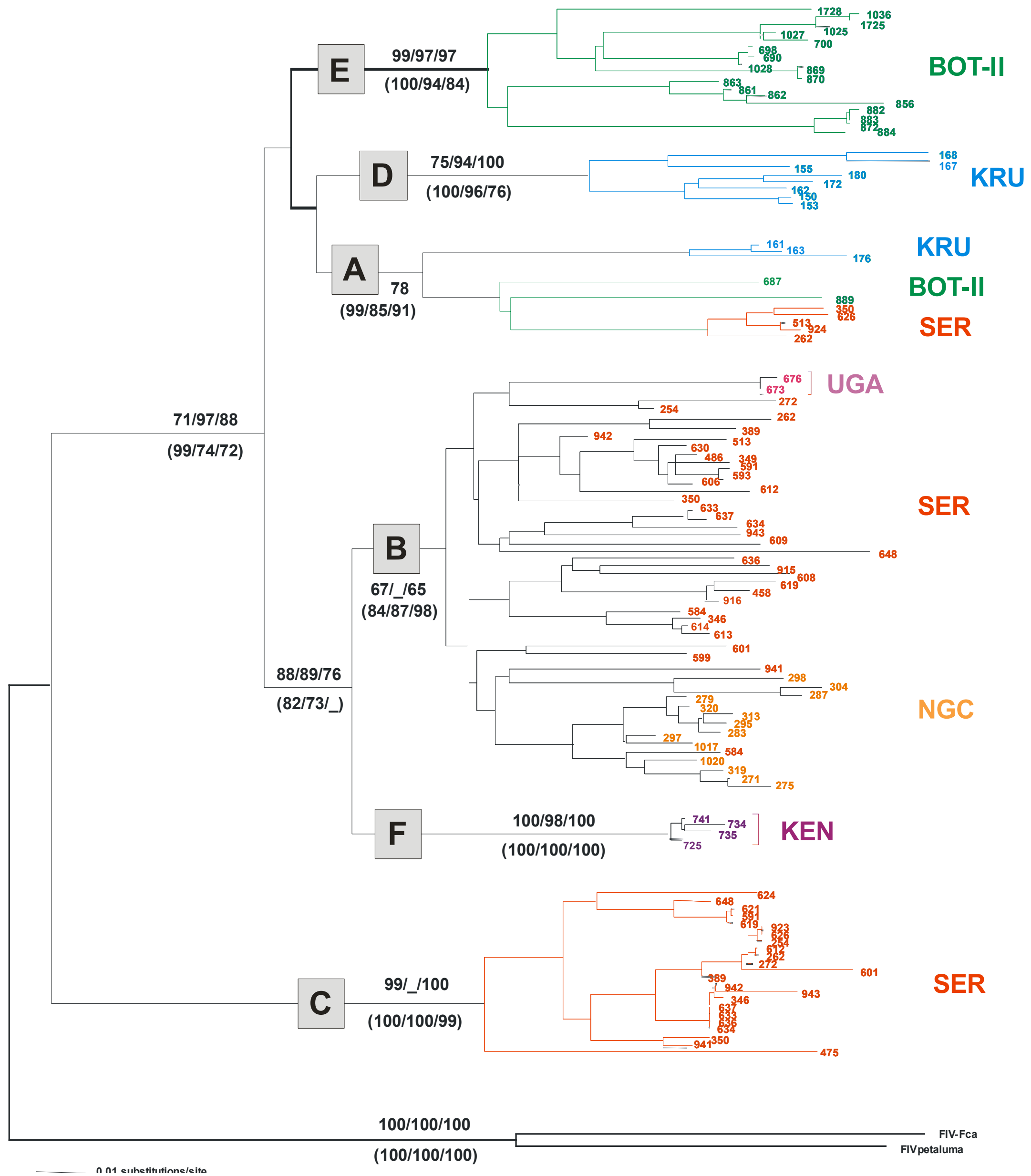

Figure S4. Phylogenetic relationships of the FIV_{Ple} *pol*-RT sequences. Neighbour-joining tree of the 301 bp FIV_{Ple} *pol*-RT sequences. The distinct FIV_{Ple} subtypes were labelled A to F. Bootstrap (BPS) values are placed at each branchpoint (ME/MP/ML) and in parenthesis are the BPS values obtained for a tree established with 520 bp of FIV_{Ple} *pol*-RT sequence for a representative subset of individuals.
