# Supplementary material for: The Evolutionary Dynamics of the Lion Panthera leo Revealed by Host and Viral Population Genomics
Source: PLoS Genet. 2008 Nov 7;4(11):e1000251. doi: 10.1371/journal.pgen.1000251 (PMC2572142; doi:10.1371/journal.pgen.1000251)
Supplement: Figure S5 — Nested design and summary results of the nested clade phylogeographic analysis (NCPA) for lion mtDNA data. (A) Nested design of the mtDNA haplotype network used for the NCPA. (B) Summary results of the NCPA. RGF/IBD - Restricted gene flow/isolation by distance. LDC/FR – long distance colonization/fragmentation. (0.10 MB PDF) [file pgen.1000251.s005.pdf]

A

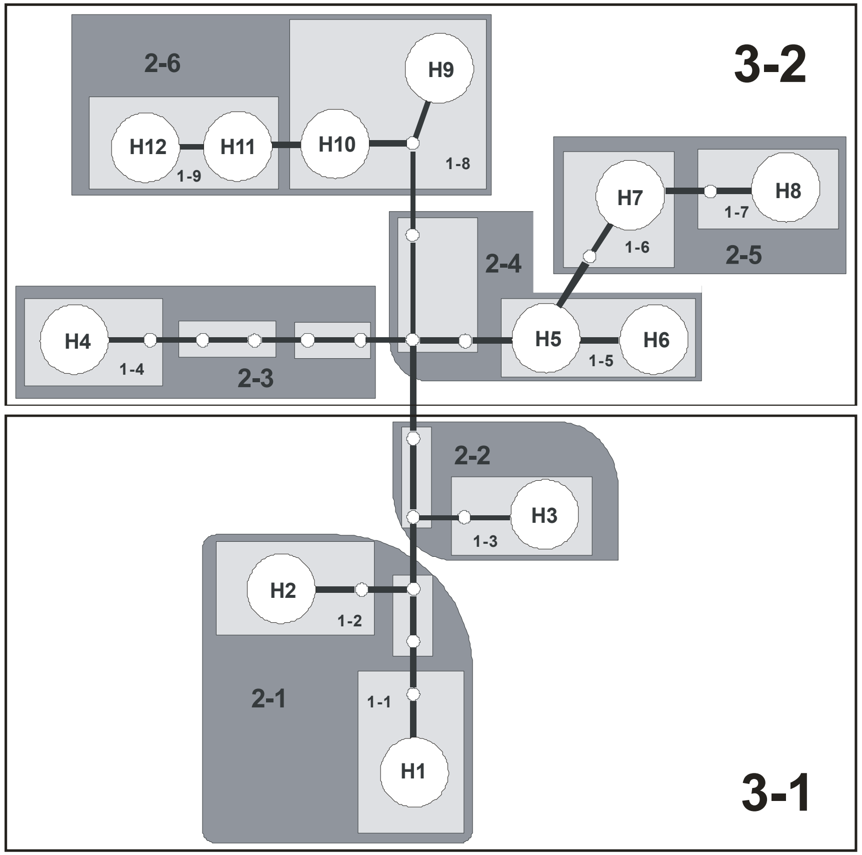

B

Clade	X^2; *P*-value	Inference
1-8	10; *P*=0.0200	RGF/IBD
2-1	71; *P*<0.0001	RGF/IBD
2-6	187.83; *P*<0.0001	RGF/IBD
2-4 and 2-5	690; *P*<0.0001	LDC/FR

Figure S5. Nested design and summary results of the nested clade phylogeographic analysis (NCPA) for lion mtDNA data. (**A**) Nested design of the mtDNA haplotype network used for the NCPA. (**B**) Summary results of the NCPA. RGF/IBD - Restricted gene flow/isolation by distance. LDC/FR – long distance colonization/fragmentation.
