# Supplementary material for: The Evolutionary Dynamics of the Lion Panthera leo Revealed by Host and Viral Population Genomics
Source: PLoS Genet. 2008 Nov 7;4(11):e1000251. doi: 10.1371/journal.pgen.1000251 (PMC2572142; doi:10.1371/journal.pgen.1000251)
Supplement: Table S1 — List of the lion samples used in this study. (0.11 MB PDF) [file pgen.1000251.s006.pdf]

Table S1. List of the lion samples used in this study.

Code Name	sex	Status	Country	Code Name	sex	Status	Country	Code Name	sex	Status	Country
PLE-ANG01	F	captive	ANGOLA	PLE-0442	M	wild	NAMIBIA	PLE-0350	M	wild	TANZANIA
PLE-ANG02	M	captive	ANGOLA	PLE-0443	M	wild	NAMIBIA	PLE-0356	F	wild	TANZANIA
PLE-0683	M	wild	BOTSWANA	PLE-0448	F	wild	NAMIBIA	PLE-0357	F	wild	TANZANIA
PLE-0685	F	wild	BOTSWANA	PLE-0984	M	wild	NAMIBIA	PLE-0358	F	wild	TANZANIA
PLE-0686	F	wild	BOTSWANA	PLE-0986	F	wild	NAMIBIA	PLE-0360	F	wild	TANZANIA
PLE-0687	M	wild	BOTSWANA	PLE-0989	M	wild	NAMIBIA	PLE-0361	F	wild	TANZANIA
PLE-0690	M	wild	BOTSWANA	PLE-0992	F	wild	NAMIBIA	PLE-0364	M	wild	TANZANIA
PLE-0691	F	wild	BOTSWANA	PLE-0993	M	wild	NAMIBIA	PLE-0365	M	wild	TANZANIA
PLE-0692	M	wild	BOTSWANA	PLE-0994	M	wild	NAMIBIA	PLE-0372	F	wild	TANZANIA
PLE-0693	F	wild	BOTSWANA	PLE-0995	F	wild	NAMIBIA	PLE-0377	F	wild	TANZANIA
PLE-0694	F	wild	BOTSWANA	PLE-0996	M	wild	NAMIBIA	PLE-0382	M	wild	TANZANIA
PLE-0695	F	wild	BOTSWANA	PLE-0999	F	wild	NAMIBIA	PLE-0384	M	wild	TANZANIA
PLE-0696	F	wild	BOTSWANA	PLE-1002	M	wild	NAMIBIA	PLE-0388	F	wild	TANZANIA
PLE-0697	F	wild	BOTSWANA	PLE-1003	M	wild	NAMIBIA	PLE-0389	F	wild	TANZANIA
PLE-0698	F	wild	BOTSWANA	PLE-1006	M	wild	NAMIBIA	PLE-0390	F	wild	TANZANIA
PLE-0699	F	wild	BOTSWANA	PLE-1007	F	wild	NAMIBIA	PLE-0391	F	wild	TANZANIA
PLE-0700	F	wild	BOTSWANA	PLE-1009	F	wild	NAMIBIA	PLE-0392	F	wild	TANZANIA
PLE-0855	F	wild	BOTSWANA	PLE-1010	M	wild	NAMIBIA	PLE-0458	F	wild	TANZANIA
PLE-0856	F	wild	BOTSWANA	PLE-1011	F	wild	NAMIBIA	PLE-0465	F	wild	TANZANIA
PLE-0857	F	wild	BOTSWANA	PLE-0035	M	captive	SOUTH AFRICA	PLE-0466	F	wild	TANZANIA
PLE-0859	M	wild	BOTSWANA	PLE-0036	F	captive	SOUTH AFRICA	PLE-0467	F	wild	TANZANIA
PLE-0860	F	wild	BOTSWANA	PLE-0150	M	wild	SOUTH AFRICA	PLE-0468	F	wild	TANZANIA
PLE-0861	F	wild	BOTSWANA	PLE-0153	M	wild	SOUTH AFRICA	PLE-0469	F	wild	TANZANIA
PLE-0862	F	wild	BOTSWANA	PLE-0155	M	wild	SOUTH AFRICA	PLE-0471	F	wild	TANZANIA
PLE-0863	F	wild	BOTSWANA	PLE-0157	F	wild	SOUTH AFRICA	PLE-0472	F	wild	TANZANIA
PLE-0869	Unknown	wild	BOTSWANA	PLE-0161	F	wild	SOUTH AFRICA	PLE-0473	M	wild	TANZANIA
PLE-0870	Unknown	wild	BOTSWANA	PLE-0162	F	wild	SOUTH AFRICA	PLE-0474	F	wild	TANZANIA
PLE-0872	F	wild	BOTSWANA	PLE-0163	M	wild	SOUTH AFRICA	PLE-0476	M	wild	TANZANIA
PLE-0873	M	wild	BOTSWANA	PLE-0167	F	wild	SOUTH AFRICA	PLE-0481	M	wild	TANZANIA
PLE-0875	M	wild	BOTSWANA	PLE-0168	M	captive	SOUTH AFRICA	PLE-0482	M	wild	TANZANIA
PLE-0876	F	wild	BOTSWANA	PLE-0171	Unknown	wild	SOUTH AFRICA	PLE-0483	F	wild	TANZANIA
PLE-0877	M	wild	BOTSWANA	PLE-0172	M	wild	SOUTH AFRICA	PLE-0484	F	wild	TANZANIA
PLE-0880	F	wild	BOTSWANA	PLE-0173	M	wild	SOUTH AFRICA	PLE-0485	F	wild	TANZANIA
PLE-0881	F	wild	BOTSWANA	PLE-0175	M	wild	SOUTH AFRICA	PLE-0486	F	wild	TANZANIA
PLE-0882		wild	BOTSWANA	PLE-0176	F	wild	SOUTH AFRICA	PLE-0487	F	wild	TANZANIA
PLE-0883	F	wild	BOTSWANA	PLE-0177	F	wild	SOUTH AFRICA	PLE-0489	F	wild	TANZANIA
PLE-0884	M	wild	BOTSWANA	PLE-0179	M	wild	SOUTH AFRICA	PLE-0490	F	wild	TANZANIA
PLE-0885	M	wild	BOTSWANA	PLE-0180	M	wild	SOUTH AFRICA	PLE-0494	M	wild	TANZANIA
PLE-0889	M	wild	BOTSWANA	PLE-0181	Unknown	wild	SOUTH AFRICA	PLE-0502	M	wild	TANZANIA
PLE-0893	Unknown	wild	BOTSWANA	PLE-0701	M	wild	SOUTH AFRICA	PLE-0503	F	wild	TANZANIA
PLE-0894	Unknown	wild	BOTSWANA	PLE-0702	M	wild	SOUTH AFRICA	PLE-0504	M	wild	TANZANIA
PLE-0895	Unknown	wild	BOTSWANA	PLE-0703	M	wild	SOUTH AFRICA	PLE-0505	M	wild	TANZANIA
PLE-0896	F	wild	BOTSWANA	PLE-0704	F	wild	SOUTH AFRICA	PLE-0507	M	wild	TANZANIA
PLE-0897	F	wild	BOTSWANA	PLE-0705	F	wild	SOUTH AFRICA	PLE-0508	M	wild	TANZANIA
PLE-0898	Unknown	wild	BOTSWANA	PLE-0706	F	wild	SOUTH AFRICA	PLE-0511	F	wild	TANZANIA
PLE-0899	Unknown	wild	BOTSWANA	PLE-0707	M	wild	SOUTH AFRICA	PLE-0512	F	wild	TANZANIA
PLE-0900	Unknown	wild	BOTSWANA	PLE-0708	M	wild	SOUTH AFRICA	PLE-0513	F	wild	TANZANIA
PLE-0902	M	wild	BOTSWANA	PLE-0709	M	wild	SOUTH AFRICA	PLE-0515	M	wild	TANZANIA
PLE-0903	Unknown	wild	BOTSWANA	PLE-0710	F	wild	SOUTH AFRICA	PLE-0517	M	wild	TANZANIA
PLE-0905	Unknown	wild	BOTSWANA	PLE-0711	F	wild	SOUTH AFRICA	PLE-0518	M	wild	TANZANIA
PLE-0971	F	wild	BOTSWANA	PLE-0712	M	wild	SOUTH AFRICA	PLE-0526	F	wild	TANZANIA
PLE-0975	M	wild	BOTSWANA	PLE-0713	F	wild	SOUTH AFRICA	PLE-0528	M	wild	TANZANIA
PLE-1025	F	wild	BOTSWANA	PLE-0714	F	wild	SOUTH AFRICA	PLE-0529	M	wild	TANZANIA
PLE-1026	M	wild	BOTSWANA	PLE-0715	M	wild	SOUTH AFRICA	PLE-0532	F	wild	TANZANIA
PLE-1027	M	wild	BOTSWANA	PLE-0716	F	wild	SOUTH AFRICA	PLE-0534	M	wild	TANZANIA
PLE-1028	F	wild	BOTSWANA	PLE-0717	M	wild	SOUTH AFRICA	PLE-0547	Unknown	wild	TANZANIA
PLE-1029	F	wild	BOTSWANA	PLE-0718	M	wild	SOUTH AFRICA	PLE-0554	M	wild	TANZANIA
PLE-1030	F	wild	BOTSWANA	PLE-0720	F	wild	SOUTH AFRICA	PLE-0567	F	wild	TANZANIA
PLE-1031	F	wild	BOTSWANA	PLE-0721	M	wild	SOUTH AFRICA	PLE-0570	F	wild	TANZANIA

Table S1. Continued

Code Name	sex	Status	Country	Code Name	sex	Status	Country	Code Name	sex	Status	Country
PLE-1032	F	wild	BOTSWANA	PLE-0722	M	wild	SOUTH AFRICA	PLE-0579	F	wild	TANZANIA
PLE-1034	F	wild	BOTSWANA	PLE-0723	F	wild	SOUTH AFRICA	PLE-0584	M	wild	TANZANIA
PLE-1035	M	wild	BOTSWANA	PLE-0724	M	wild	SOUTH AFRICA	PLE-0585	M	wild	TANZANIA
PLE-1036	M	wild	BOTSWANA	PLE-1045	F	captive	SOUTH AFRICA	PLE-0586	F	wild	TANZANIA
PLE-1037	F	wild	BOTSWANA	PLE-0227	M	wild	TANZANIA	PLE-0587	M	wild	TANZANIA
PLE-1039	F	wild	BOTSWANA	PLE-0228	M	wild	TANZANIA	PLE-0588	M	wild	TANZANIA
PLE-1725	F	wild	BOTSWANA	PLE-0229	M	wild	TANZANIA	PLE-0590	F	wild	TANZANIA
PLE-1726	F	wild	BOTSWANA	PLE-0230	M	wild	TANZANIA	PLE-0591	M	wild	TANZANIA
PLE-1728	M	wild	BOTSWANA	PLE-0242	F	wild	TANZANIA	PLE-0592	M	wild	TANZANIA
PLE-1729	F	wild	BOTSWANA	PLE-0243	F	wild	TANZANIA	PLE-0593	F	wild	TANZANIA
PLE-1731	M	wild	BOTSWANA	PLE-0246	F	wild	TANZANIA	PLE-0598	F	wild	TANZANIA
PLE-1734	F	wild	BOTSWANA	PLE-0247	F	wild	TANZANIA	PLE-0599	F	wild	TANZANIA
PLE-0050	F	captive	INDIA	PLE-0248	F	wild	TANZANIA	PLE-0600	M	wild	TANZANIA
PLE-0053	F	captive	INDIA	PLE-0249	F	wild	TANZANIA	PLE-0601	F	wild	TANZANIA
PLE-0058	F	captive	INDIA	PLE-0250	F	wild	TANZANIA	PLE-0606	F	wild	TANZANIA
PLE-0060	M	captive	INDIA	PLE-0251	F	wild	TANZANIA	PLE-0608	F	wild	TANZANIA
PLE-0067	F	captive	INDIA	PLE-0252	F	wild	TANZANIA	PLE-0609	F	wild	TANZANIA
PLE-0068	F	captive	INDIA	PLE-0253	M	wild	TANZANIA	PLE-0612	M	wild	TANZANIA
PLE-0069	F	captive	INDIA	PLE-0254	F	wild	TANZANIA	PLE-0613	M	wild	TANZANIA
PLE-0070	F	captive	INDIA	PLE-0255	F	wild	TANZANIA	PLE-0614	F	wild	TANZANIA
PLE-0071	M	captive	INDIA	PLE-0257	F	wild	TANZANIA	PLE-0616	F	wild	TANZANIA
PLE-0202	M	captive	INDIA	PLE-0258	M	wild	TANZANIA	PLE-0617	M	wild	TANZANIA
PLE-0205	F	captive	INDIA	PLE-0259	F	wild	TANZANIA	PLE-0619	M	wild	TANZANIA
PLE-0725	M	wild	KENYA	PLE-0261	M	wild	TANZANIA	PLE-0621	F	wild	TANZANIA
PLE-0734	F	wild	KENYA	PLE-0262	F	wild	TANZANIA	PLE-0622	F	wild	TANZANIA
PLE-0735	M	wild	KENYA	PLE-0265	F	wild	TANZANIA	PLE-0624	M	wild	TANZANIA
PLE-0736	M	wild	KENYA	PLE-0266	F	wild	TANZANIA	PLE-0626	F	wild	TANZANIA
PLE-0741	M	wild	KENYA	PLE-0267	F	wild	TANZANIA	PLE-0628	F	wild	TANZANIA
PLE-0742	F	wild	KENYA	PLE-0270	F	wild	TANZANIA	PLE-0630	F	wild	TANZANIA
PLE-0743	M	wild	KENYA	PLE-0271	F	wild	TANZANIA	PLE-0633	F	wild	TANZANIA
PLE-0744	F	wild	KENYA	PLE-0272	F	wild	TANZANIA	PLE-0634	F	wild	TANZANIA
PLE-0745	M	wild	KENYA	PLE-0275	F	wild	TANZANIA	PLE-0635	F	wild	TANZANIA
PLE-0748	M	wild	KENYA	PLE-0279	M	wild	TANZANIA	PLE-0636	F	wild	TANZANIA
PLE-0750	F	wild	KENYA	PLE-0283	F	wild	TANZANIA	PLE-0637	F	wild	TANZANIA
PLE-0763	F	wild	KENYA	PLE-0287	M	wild	TANZANIA	PLE-0638	M	wild	TANZANIA
PLE-0766	F	wild	KENYA	PLE-0295	F	wild	TANZANIA	PLE-0648	M	wild	TANZANIA
PLE-0005	F	captive	MOROCCO	PLE-0297	F	wild	TANZANIA	PLE-0913	F	wild	TANZANIA
PLE-0006	F	captive	MOROCCO	PLE-0298	F	wild	TANZANIA	PLE-0914	F	wild	TANZANIA
PLE-0007	F	captive	MOROCCO	PLE-0304	F	wild	TANZANIA	PLE-0915	M	wild	TANZANIA
PLE-0666	M	captive	MOROCCO	PLE-0305	F	wild	TANZANIA	PLE-0916	F	wild	TANZANIA
PLE-0401	F	wild	NAMIBIA	PLE-0311	F	wild	TANZANIA	PLE-0919	M	wild	TANZANIA
PLE-0402	F	wild	NAMIBIA	PLE-0313	M	wild	TANZANIA	PLE-0922	F	wild	TANZANIA
PLE-0403	M	wild	NAMIBIA	PLE-0315	F	wild	TANZANIA	PLE-0923	F	wild	TANZANIA
PLE-0404	F	wild	NAMIBIA	PLE-0317	M	wild	TANZANIA	PLE-0924	M	wild	TANZANIA
PLE-0405	M	wild	NAMIBIA	PLE-0319	M	wild	TANZANIA	PLE-0941	F	wild	TANZANIA
PLE-0409	M	wild	NAMIBIA	PLE-0320	M	wild	TANZANIA	PLE-0942	M	wild	TANZANIA
PLE-0410	M	wild	NAMIBIA	PLE-0326	M	wild	TANZANIA	PLE-0943	M	wild	TANZANIA
PLE-0411	F	wild	NAMIBIA	PLE-0327	M	wild	TANZANIA	PLE-1016	M	wild	TANZANIA
PLE-0412	M	wild	NAMIBIA	PLE-0328	F	wild	TANZANIA	PLE-1017	F	wild	TANZANIA
PLE-0413	F	wild	NAMIBIA	PLE-0330	F	wild	TANZANIA	PLE-1020	F	wild	TANZANIA
PLE-0415	F	wild	NAMIBIA	PLE-0331	M	wild	TANZANIA	PLE-0029	M	captive	ZIMBABWE
PLE-0416	M	wild	NAMIBIA	PLE-0332	M	wild	TANZANIA	PLE-0668	F	wild	UGANDA
PLE-0417	F	wild	NAMIBIA	PLE-0333	M	wild	TANZANIA	PLE-0669	M	wild	UGANDA
PLE-0418	F	wild	NAMIBIA	PLE-0334	M	wild	TANZANIA	PLE-0670	M	wild	UGANDA
PLE-0419	M	wild	NAMIBIA	PLE-0335	M	wild	TANZANIA	PLE-0671	M	wild	UGANDA
PLE-0422	F	wild	NAMIBIA	PLE-0338	M	wild	TANZANIA	PLE-0672	F	wild	UGANDA
PLE-0425	M	wild	NAMIBIA	PLE-0345	M	wild	TANZANIA	PLE-0673	M	wild	UGANDA
PLE-0427	M	wild	NAMIBIA	PLE-0346	M	wild	TANZANIA	PLE-0674	M	wild	UGANDA
PLE-0437	M	wild	NAMIBIA	PLE-0347	M	wild	TANZANIA	PLE-0675	F	wild	UGANDA
PLE-0439	M	wild	NAMIBIA	PLE-0349	M	wild	TANZANIA	PLE-0676	F	wild	UGANDA

BOTSWANA (Chobe National Park, Makgadikgadi National Park, Mpathutlwa Pan, Monamodi Pan, Nxai Pan, Okavango, Vumbora, Kwando, Whater Cut); INDIA (Gir Forest); KENYA (Laikipia); NAMÍBIA (Charitsaub, Duikerdrink, Eindpaal, Gemsbokvlakte, Gobaub, M’Bari, Okaukuejo, Okondeka, Skeleton Coast, West Etosha National Park); SOUTH AFRICA (South Kalahari, Kruger National Park); TANZANIA (Ngorongoro Crater, Serengeti National Park); UGANDA (Queen Elizabeth National Park).
