# Supplementary material for: The Evolutionary Dynamics of the Lion Panthera leo Revealed by Host and Viral Population Genomics
Source: PLoS Genet. 2008 Nov 7;4(11):e1000251. doi: 10.1371/journal.pgen.1000251 (PMC2572142; doi:10.1371/journal.pgen.1000251)
Supplement: Table S2 — Primers used to amplify the mtDNA (12S–16S) and nDNA (ADA and TF) portions surveyed in this study. (0.06 MB PDF) [file pgen.1000251.s007.pdf]

Table S2. Primers used to amplify the mtDNA (*12S-16S*) and nDNA (*ADA* and *TF*) portions surveyed in this study.

**Primer Name**	**Sequence**
mtDNA (*12S-16S*)	
O16S-CY-R	TTTTGGATCAATAAGTG
L12S-CY-F	CTTAAGTGACTAGCCCCTA
PAN-16S-CY-R	CAGAACTCAGATCACGTAG
12S-UP-F	AAAAAGCTTCAAACTGGGATTAGATACCCCACTAT
ADA (exon 10 and intron 10)	
AA-ADA-ex10-F	GACCCGCTCATCTTCAAGTC
AA-ADA-ex11-R	CCCGTAGGCCTTATAGAGCA
TF (intron 3)	
AA-TF-ex3-F	GTGACGTTGGATGCAGGTTTGGTG
AA-TF-ex4-R	CAATGGGGATGTTCCACCCAGC
