# Supplementary material for: The Evolutionary Dynamics of the Lion Panthera leo Revealed by Host and Viral Population Genomics
Source: PLoS Genet. 2008 Nov 7;4(11):e1000251. doi: 10.1371/journal.pgen.1000251 (PMC2572142; doi:10.1371/journal.pgen.1000251)
Supplement: Table S3 — Structure cluster assignment results of 357 lions based on nDNA (ADA, TF, and 22 microsatellites) and mtDNA markers. Burn-in and replication values set at 30,000 and 1,000,000, respectively. (0.06 MB PDF) [file pgen.1000251.s008.pdf]

Table S3. Structure cluster assignment results of 357 lions based on nDNA (*ADA* , *TF* , and 22 microsatellites) and mtDNA markers. Burn-in and replication values set at 30,000 and 1,000,000, respectively.

K	Ln	Var[LnP(D)]	alpha	Pr(K)
1	-17517.8	105.9	-	0
2	-16378.0	335.9	0.0862	0
3	-15690.2	422.4	0.0425	0
4	-15197.2	526.5	0.0392	0
5	-14872.4	550.6	0.0315	0
6	-14719.1	678.6	0.0325	0
7	-14555.4	811.5	0.0338	~0
8	-14297.3	902.2	0.0325	~0
9	-14430.1	1368.2	0.0325	~0
10	-14171.6	1116.0	0.0318	~0
12	-14762.5	2916.6	0.0329	0
13	-14373.8	2335.9	0.0330	~0
14	-14016.4	1820.1	0.0332	~0
