# Supplementary material for: The Evolutionary Dynamics of the Lion Panthera leo Revealed by Host and Viral Population Genomics
Source: PLoS Genet. 2008 Nov 7;4(11):e1000251. doi: 10.1371/journal.pgen.1000251 (PMC2572142; doi:10.1371/journal.pgen.1000251)
Supplement: Table S4 — Gene diversity and frequency values in lion populations. (0.07 MB PDF) [file pgen.1000251.s009.pdf]

Table S4. Gene diversity and frequency values in lion populations.

	Haplotype data						*Frequency data*								
	mtDNA		*nDNA*				*nDNA*								
	12S and *16S*		*ADA*		*TF*		*microsatellites*					*ADA + TF + microsatellites*			
Population	*h*	*π*	*h*	*π*	*h*	*π*	H_E	H_O	P_{95}	*A*	A_U	H_E	H_O	P_{95}	*A*
GIR	0.53	0.00057	_	_	_	_	0.106	0.122	0.32	1.4	3	0.097	0.112	0.29	1.4
UGA	_	_	_	_	0.30	0.00359	0.471	0.621	0.86	2.8	3	0.442	0.582	0.83	2.7
KEN	_	_	0.66	0.00268	0.67	0.00742	0.692	0.684	1	5.1	5	0.688	0.679	1	5.0
SER-I	0.03	0.00001	0.45	0.00208	0.51	0.00616	0.643	0.637	1	6.0	2	0.628	0.623	1	5.7
SER-II	_	_	0.40	0.00165	0.56	0.00620	0.596	0.653	1	5.1	5	0.586	0.637	1	5.0
SER-III	_	_	0.28	0.00113	0.52	0.00586	0.640	0.667	1	5.7	4	0.620	0.641	1	5.5
NGC	_	_	0.28	0.00120	0.48	0.00569	0.586	0.613	1	4.2	0	0.569	0.588	1	4.0
KRU	0.41	0.00151	0.23	0.00107	0.32	0.00382	0.574	0.649	0.95	4.2	4	0.546	0.619	0.96	4.0
BOT-I	_	_	0.24	0.00110	0.49	0.00584	0.628	0.602	1	4.8	5	0.606	0.586	1	4.6
BOT-II	0.29	0.00109	0.08	0.00038	0.62	0.00737	0.699	0.736	1	5.7	5	0.668	0.704	0.96	5.4
NAM	0.21	0.00056	0.51	0.00238	0.50	0.00582	0.572	0.577	1	4.4	4	0.565	0.570	1	4.2

h - haplotype diversity
π - nucleotide diversity
H_E - expected heterozygosity
H_O - observed heterozygosity
P_{95} - percentage of polymorphic loci at 5%
A - mean number of alleles
A_U - number of unique alleles
