# Supplementary material for: The Evolutionary Dynamics of the Lion Panthera leo Revealed by Host and Viral Population Genomics
Source: PLoS Genet. 2008 Nov 7;4(11):e1000251. doi: 10.1371/journal.pgen.1000251 (PMC2572142; doi:10.1371/journal.pgen.1000251)
Supplement: Table S5 — Bottleneck analysis in lion populations using the standardized differences test and the stepwise mutation model (SMM). (0.05 MB PDF) [file pgen.1000251.s010.pdf]

Table S5. Bottleneck analysis in lion populations using the standardized differences test and the stepwise mutation model (SMM).

	Standardized differences test
	SMM
GIR	*
UGA	ns
KEN	ns
SER-I	***
SER-II	**
SER-III	***
NGC	*
KRU	*
BOT-I	*
BOT-II	ns
NAM	ns

ns - not significant; *P<0.05; **P<0.01; ***P<0.001.
