# Supplementary material for: The Evolutionary Dynamics of the Lion Panthera leo Revealed by Host and Viral Population Genomics
Source: PLoS Genet. 2008 Nov 7;4(11):e1000251. doi: 10.1371/journal.pgen.1000251 (PMC2572142; doi:10.1371/journal.pgen.1000251)
Supplement: Table S6 — Results of the hierarchical AMOVA in lions for four different geographical scenarios. (0.06 MB PDF) [file pgen.1000251.s011.pdf]

Table S6. Results of the hierarchical AMOVA in lions for four different geographical scenarios.

	3-group scenario[a]		4-group scenario[b]		5-group scenario[c]		6-group scenario[d]	
	Φ statistics	% total variation	Φ statistics	% total variation	Φ statistics	% total variation	Φ statistics	% total variation
within-population	Φ_{ST} = 0.916***	8.43	Φ_{ST} = 0.916***	8.38	Φ_{ST} = 0.916***	8.44	Φ_{ST} = 0.916***	8.40
among-population within-groups	Φ_{SC} = 0.729***	22.67	Φ_{SC} = 0.476***	7.62	Φ_{SC} = 0.192**	2.00	Φ_{SC} = 0.032^{NS}	0.28
among-groups	Φ_{CT} = 0.689*	68.91	Φ_{CT} = 0.840***	84.00	Φ_{CT} = 0.896***	89.55	Φ_{CT} = 0.913***	91.32

[a] 3-group scenario = [(ANG+ATL+GIR), (UGA+KEN+SER+NGC+BOT-I), (NAM,BOT-II, KRU)]
[b] 4-group scenario = [(ANG+ATL+GIR), (UGA+SER+NGC+BOT-I), (KEN), (NAM,BOT-II, KRU)]
[c] 5-group scenario = [(ANG+ATL+GIR), (UGA+SER+NGC+BOT-I), (KEN), (NAM,BOT-II), (KRU)]
[d] 6-group scenario = [(ANG+ATL), (GIR), (UGA+SER+NGC+BOT-I), (KEN), (NAM,BOT-II), (KRU)]
NS - not significant; * P < 0.05; ** P < 0.001; *** P < 0.0001.
