# Supplementary material for: The Evolutionary Dynamics of the Lion Panthera leo Revealed by Host and Viral Population Genomics
Source: PLoS Genet. 2008 Nov 7;4(11):e1000251. doi: 10.1371/journal.pgen.1000251 (PMC2572142; doi:10.1371/journal.pgen.1000251)
Supplement: Table S7 — Lion population pairwise F ST estimates. Below the diagonal mtDNA data (12S–16S) and above the diagonal microsatellite data (22 loci). (0.06 MB PDF) [file pgen.1000251.s012.pdf]

Table S7. Lion population pairwise $F_{ST}$ estimates. Below the diagonal mtDNA data (*12S-16S*) and above the diagonal microsatellite data (22 loci).

| Populations | | | | | | | | | | | |
|---|---|---|---|---|---|---|---|---|---|---|---|
| | GIR | UGA | KEN | NGC | SER-I | SER-II | SER-III | KRU | BOT-I | BOT-II | NAM |
| GIR | _ | 0.59490 | 0.42296 | 0.51355 | 0.42389 | 0.45582 | 0.42903 | 0.53709 | 0.42459 | 0.39594 | 0.47708 |
| UGA | 0.92157 | _ | 0.13685 | 0.22677 | 0.18111 | 0.23260 | 0.18323 | 0.24310 | 0.20616 | 0.17590 | 0.30731 |
| KEN | 0.94667 | 1 | _ | 0.15719 | 0.08134 | 0.11455 | 0.09455 | 0.13799 | 0.13843 | 0.09341 | 0.20509 |
| NGC | 0.92157 | _ | 1 | _ | 0.11771 | 0.15108 | 0.11405 | 0.26699 | 0.18085 | 0.14562 | 0.26318 |
| SER-I | 0.91677 | 0.01852 | 0.99644 | 0.01852 | _ | 0.05463 | 0.04137 | 0.13276 | 0.13428 | 0.11136 | 0.22015 |
| SER-II | 0.92157 | _ | 1 | _ | 0.01852 | _ | 0.06458 | 0.18868 | 0.13298 | 0.14046 | 0.25026 |
| SER-III | 0.92157 | _ | 1 | _ | 0.01852 | _ | _ | 0.16163 | 0.12167 | 0.09745 | 0.20037 |
| KRU | 0.74231 | 0.77174 | 0.84783 | 0.77174 | 0.76789 | 0.77174 | 0.77174 | _ | 0.14698 | 0.14598 | 0.20365 |
| BOT-I | 0.92157 | _ | 1 | _ | 0.01852 | _ | _ | 0.77174 | _ | 0.09483 | 0.17988 |
| BOT-II | 0.79987 | 0.84950 | 0.88485 | 0.84950 | 0.84608 | 0.84950 | 0.84950 | 0.52011 | 0.84950 | _ | 0.14103 |
| NAM | 0.88021 | 0.93480 | 0.94588 | 0.93480 | 0.93141 | 0.93480 | 0.93480 | 0.62125 | 0.93480 | 0.06596 | _ |

*Note - the mtDNA genetic distance among African populations can be higher then between African and Asian ones.*
