# Supplementary material for: The Evolutionary Dynamics of the Lion Panthera leo Revealed by Host and Viral Population Genomics
Source: PLoS Genet. 2008 Nov 7;4(11):e1000251. doi: 10.1371/journal.pgen.1000251 (PMC2572142; doi:10.1371/journal.pgen.1000251)
Supplement: Table S8 — Taxon specific unique nDNA alleles in lion populations (FCA-microsatellites and ADA locus). (0.06 MB PDF) [file pgen.1000251.s013.pdf]

Table S8. Taxon specific unique nDNA alleles in lion populations (FCA-microsatellites and *ADA* locus).

Taxon	Locus	allele
GIR	FCA211	142
GIR	FCA14	154
GIR	FCA230	82
UGA	FCA8	137
UGA	FCA98	90
UGA	FCA230	98
KEN	FCA105	219
KEN	FCA281	261
KEN	FCA69	85
KEN	FCA14	166
KEN	FCA85	129
SER-I	FCA6	189
SER-I	FCA98	116
SER-II	FCA6	197
SER-II	**ADA**	**A3**
SER-II	FCA208	303
SER-II	FCA230	74
SER-II	FCA205	106
SER-III	FCA69	95
SER-III	FCA208	323
SER-III	FCA208	325
SER-III	FCA247	163
KRU	FCA211	154
KRU	FCA98	122
KRU	FCA247	161
KRU	FCA205	118
BOT-I	FCA281	236
BOT-I	FCA281	266
BOT-I	FCA281	268
BOT-I	FCA91	164
BOT-I	FCA208	299
BOT-II	FCA8	119
BOT-II	FCA77	183
BOT-II	FCA91	162
BOT-II	FCA98	104
BOT-II	FCA129	183
NAM	FCA6	213
NAM	FCA98	118
NAM	FCA230	76
NAM	FCA205	112

Note-1: individual ATL lions showed two unique alleles (FCA8*135 and FCA8*145)

Note-2: individual ZBW lion showed one unique alleles (FCA126*175)
