# Supplementary material for: The Evolutionary Dynamics of the Lion Panthera leo Revealed by Host and Viral Population Genomics
Source: PLoS Genet. 2008 Nov 7;4(11):e1000251. doi: 10.1371/journal.pgen.1000251 (PMC2572142; doi:10.1371/journal.pgen.1000251)
Supplement: Table S9 — Summary statistics for FIVPle data. (0.00 MB PDF) [file pgen.1000251.s014.pdf]

Table S9. Summary statistics for FIV_{Ple} data.

	FIV statistics within lion populations								FIV-Ple subtypes statistics					
	UGA	KEN	NGC	SER-I	SER-II	SER-III	KRU	BOT-II	A	B	C	D	E	F
N	2	6	14	27	14	15	11	21	9	43	18	8	19	4
h	1	0.867	0.989	0.972	0.956	1	0.964	0.967	1	0.997	0.817	0.929	0.971	0.833
π	0.003	0.055	0.069	0.132	0.149	0.134	0.101	0.103	0.090	0.091	0.036	0.066	0.082	0.007
S	1	37	43	102	77	104	73	79	48	78	33	44	63	4

h - haplotype diversity
π - nucleotide diversity
S - segregrating sites
